# Investigating the Effects of Adding Explicit Teaching and/or Signing to Story‐Based Vocabulary Intervention for School‐Aged Children With (Developmental) Language Disorder

**DOI:** 10.1111/1460-6984.70317

**Published:** 2026-08-31

**Authors:** Lucy Hughes, Cécile Larralde, Hilary Nicoll, Nataša Marić, Caroline Burke, Susan Ebbels

**Affiliations:** ^1^ Moor House Research & Training Institute Moor House School & College Oxted UK; ^2^ School of Psychology and Clinical Language Sciences University of Reading Reading UK; ^3^ Division of Psychology and Language Sciences University College London London UK

**Keywords:** active ingredients, developmental language disorder, explicit intervention, school‐aged children, signing, vocabulary

## Abstract

**Background:**

Children with language disorders, including developmental language disorder (DLD), experience challenges with word learning. Common components of vocabulary interventions include presenting target words in a story, providing explicit teaching and using sign/gesture alongside speech. However, previous studies have not investigated the relative contribution of these components, in isolation or combination.

**Aim:**

To measure the effects of adding explicit teaching and/or augmentative signing to a story‐based vocabulary intervention.

**Methods:**

Eighteen participants, aged 7;09–15;02 years, took part in our within‐participant design study. All had a language disorder; 14 with DLD, and four with an associated biomedical condition. They were taught 48 real verbs and adjectives, randomly assigned to one of four conditions, over an 8‐week intervention period. In the reference condition, participants watched videos of a speech and language therapist (SLT) reading a story where target words were presented incidentally, four at a time. In the first experimental condition, the delivering SLT added explicit teaching to the reference intervention. This included: providing definitions, modelling words in sentences, requesting retrieval and employing a cueing hierarchy. The second experimental condition added augmentative signing to the reference intervention, whereby the SLT in the video signed the target words as they appeared in the story. The final experimental condition added both explicit teaching and signing. In all conditions, frequency of word presentations was controlled. Word sets were counterbalanced to minimise confounds. Outcome measures assessed vocabulary knowledge and use with: (1) a word definition task and (2) sentence generation.

**Results:**

Participants showed stronger progress in all experimental conditions, compared to the reference condition. Bayesian models showed that adding both explicit teaching and augmentative signing in combination to the reference intervention did not lead to greater progress than adding just one of these components. Neither participants’ age nor language ability were associated with intervention progress.

**Discussion:**

Our results indicate that adding either explicit teaching or augmentative signing to story‐based vocabulary intervention is similarly advantageous. Adding both does not confer additional advantage. Larger‐scale studies are needed to explore differences between participants and experimental conditions.

**Conclusions/Implications:**

Our study highlights the importance of evaluating systematically the contribution of potential active ingredients within interventions. In particular, analysing how they work together and separately is important, as adding more ingredients does not necessarily improve effectiveness. Our results inform vocabulary teaching, indicating that staff should incorporate either augmentative signing or explicit teaching whenever possible to support the vocabulary learning of those with language disorders.

**WHAT THIS PAPER ADDS:**

*What is already known on this subject*

- Children with (developmental) language disorders experience difficulties with word learning, which can affect their education and social participation. Previous research indicates that story‐based intervention, explicit teaching and, in some cases, augmentative signing can support vocabulary learning. However, many interventions combine multiple components, making it difficult to determine which specific components are active ingredients, responsible for progress.

*What this paper adds to existing knowledge*

- This study systematically compared the effects of adding explicit teaching and/or augmentative signing to a story‐based (implicit) vocabulary intervention for school‐aged children with (D)LD. Our results indicate that adding explicit teaching and/or augmentative signing can lead to greater improvements in learning new verbs and adjectives than the implicit intervention alone. Combining explicit teaching and signing does not appear to lead to additional benefit.

*What are the potential or actual clinical implications of this work?*

- SLTs and school staff working with children with (D)LD should prioritise incorporating either augmentative signing or explicit teaching strategies whenever feasible to support vocabulary development. Signing may be quicker to implement as this can be delivered alongside speech. Intervention choices could be tailored to children and staff's individual preferences and skills. Larger studies are required to explore differences between participants and experimental conditions.

## 1 Introduction

Children's vocabulary knowledge underpins their ability to learn, read, communicate, and build social relationships. Strong vocabulary skills support academic achievement (Pace et al. 2019) and social wellbeing (Ma et al. 2024). For typically‐developing children, the development of word knowledge begins gradually in infancy and progresses more rapidly across pre‐school and early school age. The average six year old knows about 10 000 words (Dockrell and Messer 2004) and is estimated to acquire around 2000–3000 new items per year throughout their childhood (Nagy and Scott 2000). Whilst early language acquisition focuses on functional, high frequency nouns, later word learning includes more abstract words, including verbs and adjectives, which may have multiple meanings and can be used in a variety of communication contexts (Muraki et al. 2025). By adolescence, learning becomes slower yet deeper, with typically‐developing children acquiring more words via reading, rather than verbal input, drawing on morphological, pragmatic and metalinguistic knowledge to support their ongoing vocabulary development (Nagy and Herman 1987).

The process of learning and retrieving words draws upon several domains of a child's developing language system. Initially, they must use phonological processing to segment smaller and larger units from the ongoing stream of speech. Then, they are required to link this information with semantic content, usually following the first exposure, to create an initial representation of a new word's form and meaning. This process has been investigated within fast‐mapping studies. For a summary, see Carey (2010). As children mature and begin to encounter a wider range of words in different contexts, they add to their semantic knowledge and develop a deeper conceptual understanding of more complex words. This process is known as ‘slow mapping’ (Carey 2010). Once children reach school age, they make use of a broader range of tools and strategies to facilitate effective vocabulary acquisition. This may include accessing explicit teaching of the meaning of words and drawing upon contextual cues to infer meaning from spoken or written stories (Nippold 2007).

Developmental language disorder (DLD) is a severe and persistent difficulty in understanding and using language, which can affect children's day‐to‐day functioning, and is diagnosed in the absence of a known associated biomedical condition (Bishop et al. 2017). DLD affects around 7.58% of children at school‐entry (around two in every classroom). A further 2.34% of children meet criteria for language disorder (LD) associated with a biomedical condition, such as autism spectrum disorder (ASD), learning disability, genetic disorders or brain injury (Norbury et al. 2016). Both DLD and wider language disorders—henceforth (D)LD—can hinder children's ability to learn and retain vocabulary, which in turn can impact literacy, especially reading comprehension, as well as their long‐term educational and social outcomes (Snowling and Hulme 2021).

Studies have identified a range of word learning differences between children with (D)LD and typically‐developing (TD) children. Storkel et al. (2017) found that children with DLD required more presentations of new words to learn them within a story context (36 vs. 12 for TD peers). Separately, Ellis Weismer and Hesketh (1996) reported that language‐impaired children need slower presentation rates, especially for spoken production. These authors also found that gestural support enhanced children's understanding of locative prepositions, particularly for those with (D)LD. Meanwhile, several studies have shown that children with DLD require more distributed presentations across several days and/or with intervening material to learn and retain words (e.g., Leonard et al. 2021).

These challenges with vocabulary learning have informed a body of research, investigating the effects of word learning aids, which may act as active ingredients to support word acquisition for children with (D)LD. An active ingredient is a component of intervention (such as a technique, procedure, method of instruction or delivery context) that helps bring about the main intended action of that intervention (Frizelle and McKean 2022). Within the word‐learning literature, potential active ingredients can either be single‐ or multi‐component. For example, explicit teaching often involves multiple components, such as provision of child‐friendly definitions, exposure to words in multiple contexts and retrieval requests. Key studies addressing potential single and multi‐component active ingredients within word‐learning interventions are summarised below.

### 1.1 Implicit Intervention

Presenting target words in a story context is an example of implicit intervention for vocabulary enrichment (Ansari et al. 2025). The theoretical rationale for this approach is that stories provide meaningful contexts to support semantic processing, while multiple and varied exposures to new words can deepen understanding and encourage cross‐situational generalisation (Roembke et al. 2023). In typical development, children infer meaning from contextual clues, through a process called quick incidental learning (Oetting et al. 1995). However, children with (D)LD may show difficulties with this form of implicit learning.

Evidence for these challenges is provided in a study by Broedelet et al. (2023), which examined cross‐situational word learning in children with DLD (*N* = 26; aged 7–9 years), compared to an age‐matched group of TD peers. Participants completed a statistical learning task where novel words and objects were presented together across trials, without indicating the correct pairings. The task examined children's ability to map words and referents through implicit exposure, without explicit teaching. While both groups learned above chance, TD children significantly outperformed those with DLD. Within the DLD group, there was no significant association between children's age or vocabulary scores and their word learning performance. Findings suggest that while children with DLD can learn implicitly, they do so less efficiently than TD peers, highlighting potential limitations in implicit learning mechanisms.

Separately, Nash and Donaldson (2005) compared word learning by children with DLD and TD children in two conditions: a storytelling context (implicit intervention), where unfamiliar target nouns were embedded within stories with accompanying picture support, and an explicit teaching context, where children were given direct verbal explanations of the target words, again with picture support. All participants showed greater gains in the explicit teaching condition, compared to the story‐based condition. However, the DLD group showed significantly poorer outcomes than chronological age controls across all outcome measures, in both word learning contexts. They showed similar performance to vocabulary age‐matched peers, who were, on average, 2.5 years younger. No details were provided regarding the response profiles of individual children and whether these varied according to age or background language scores.

### 1.2 Explicit Teaching

Explicit teaching is a multi‐component method of instruction, which can include detailed elaboration of the semantic and phonological features of words (Best et al. 2021); morphological awareness training (Glisson et al. 2025) and/or the provision of child‐friendly definitions, alongside exposure to target words in different contexts and opportunities for retrieval‐based practice through rich vocabulary instruction (Beck et al. 2013). Evidence for this latter approach includes classroom‐based or extra‐curricular studies involving children with low vocabulary and/or DLD (Dyson et al. 2018; McGregor et al. 2021).

Dyson et al. (2018) propose that explicit teaching is an important tool for supporting both typically‐developing children and those with (D)LD to learn new words, particularly verbs and adjectives. Young children typically acquire nouns more quickly and effortlessly than other word classes (Waxman et al. 2013), while those with DLD show particular difficulties with verb learning and use (Kambanaros and Grohmann 2015). According to Gentner and Boroditsky (2001), concrete objects (nouns) are easier to recognise and store in the mental lexicon, while verbs and adjectives demand more complex relational processing, making them harder for young children, or those with language difficulties, to learn and generalise. Indeed, intervention studies for children with (D)LD showed more progress on concrete nouns than verbs in 9–16 year olds (Wright et al. 2018), but no difference in 16–19 year olds between abstract nouns and verbs (Ebbels et al. 2022). Furthermore, context (such as stories) may provide relatively poor information about abstract words, which could lead to misunderstandings about a word's meaning (Beck et al. 2013).

Both Dyson et al. (2018) and McGregor et al. (2021) provide evidence that children with low vocabulary, including those with (D)LD, can benefit from targeted intervention focused on explicit vocabulary teaching. The first study used a regression discontinuity design to investigate the effectiveness of a small group vocabulary programme ‘Wonderful Words’ on word learning (including nouns, verbs and adjectives) for children with poor vocabulary knowledge. The intervention produced significant change in children's understanding of explicitly taught words, though improvements did not generalise to non‐targeted words. This lack of generalization is consistent with the studies reviewed systematically by Ansari et al. (2025).

Separately, McGregor et al. (2021) carried out a case series study involving seven children with DLD in an embedded science‐camp intervention. Rich vocabulary instruction was used to target ‘Tier Two’ science words. Tier two words are more abstract concepts, which may help students’ access curriculum texts and improve both oral and reading comprehension. Examples from this study included: *compare, data, hypothesis* and *solve*.

Following the 5‐week camp, which included approximately 27 h of science + language intervention, five children showed modest gains (of 3–6 words) in targeted vocabulary and science knowledge; two did not respond or could not complete the intervention. Older children tended to respond better to the intervention, in line with previous studies (e.g., Dockrell et al. 2007), which found age to be a positive predictor of word learning in response to explicit intervention.

It is difficult to identify the mechanisms of change for the above rich vocabulary interventions, since these programmes include multiple components or techniques, such as frequent repetition of words, semantic and phonological feature analysis and multi‐sensory learning, as well as explicit definitions.

### 1.3 Shared Interactive Book Reading

Among the most widely‐used paradigms for teaching vocabulary to young children with (D)LD is shared interactive book reading (SIBR; Towson et al. 2021). This approach combines the use of implicit story contexts and explicit teaching of new words and can incorporate a range of intervention components. For example, Storkel et al. (2017) and (2019) introduced target words (a mixture of nouns, verbs and adjectives) to pre‐schoolers with DLD during a pre‐book‐reading activity. Children were shown a picture illustration and were provided with a synonym, then a child‐friendly definition of each word. Once all six target words had been previewed, the book was read to the child. Following this, the target words were reviewed, again with picture support. The child was given an example of the word being used within a sentence, and the child‐friendly definition was repeated.

Towson et al. (2021) concludes that SIBR is an effective, evidence‐based practice for young children who are typically developing and for those with developmental disabilities, including (D)LD. However, Storkel et al. (2017) and (2019) note that children's response to intervention may be linked to their underlying language profiles. Both studies found that children with lower scores on formal phonological and/or linguistic assessments made less progress following the SIBR programme. Storkel et al. (2019) also noted that children in the study showed rapid forgetting of newly‐taught words when they were retested 2 weeks after the intervention.

### 1.4 Augmentative Signing

The final potential active ingredient to be considered in this paper is the use of augmentative signing, alongside speech, to support word acquisition and retrieval in children with DLD. According to the dual coding theory (Li et al. 2022), learning and retention of vocabulary are enhanced when information is encoded in both verbal and visual forms. This multimodal representation is hypothesised to strengthen memory traces and aid children's access to words by creating two connected retrieval pathways (Goldin‐Meadow and Alibali 2013). Empirical support for the role of signing and gesture in typical word learning includes studies focusing on the role of gestures in synchronising children's attention with key features of spoken input (Wakefield et al. 2018); using embodiment (requiring children to gesture while learning a new concept) to help them represent and store new ideas (Cook et al. 2008) and conveying nuances of meaning that are not present in the accompanying speech (Novack and Goldin‐Meadow 2015).

Several studies have investigated the impact of augmentative signing on word learning for children with DLD (Bragard and Schelstraete 2023; Lüke et al. 2020; Weismer and Hesketh 1993). van Berkel‐van Hoof et al. (2019) reported that children with DLD (*N* = 40, ages 9–11) learned pseudowords significantly more accurately and quickly when iconic signs (those which capture the meaning of a word through hand shape or positioning, e.g. ‘drink’) accompanied spoken stimuli. In contrast, typically developing children showed no significant benefits from the sign‐supported approach. However, a previous study by the same authors (van Berkel‐van Hoof et al. 2016) did not find an effect of signing on vocabulary learning for children with DLD, despite the same methods and materials being used. The authors suggest that this was due to participants with DLD in the earlier study showing relatively strong receptive language, while the follow‐up study included more children with a wider range of language profiles.

Separately, Bragard and Schelstrate (2023) found both children with DLD (aged 5–10 years) and typically‐developing controls benefited from gestures to support their learning of novel names for common concepts, for example, /di:tə/ for ‘motorbike’. No differences were found in the effects of iconic versus ‘arbitrary’ gestures, lending support to theoretical accounts that adding gesture or sign may facilitate word learning by increasing children's attention to spoken input (Wakefield et al. 2018). However, items targeted in this study were already known to children; reducing the need to highlight semantic properties of the taught words. Results from the above studies indicate that further investigation is needed to establish the role of signing as a potential active ingredient to enhance receptive and/or expressive vocabulary.

### 1.5 Summary

Research on vocabulary interventions for children with (D)LD highlights the effectiveness of explicit teaching approaches; combining implicit story contexts with explicit word teaching; and the potential benefits of using augmentative signs to support word learning. However, the variability of methods and tendency to combine different components within vocabulary enrichment programmes make it challenging to identify which specific mechanisms of change may drive intervention effectiveness. This highlights the need to identify the active ingredients of vocabulary interventions and understand how these may work together or separately to influence outcomes for children with (D)LD.

### 1.6 Research Questions

In this study, we investigated the effects of adding explicit teaching and/or augmentative signing to an implicit, story‐based vocabulary intervention, teaching verbs and adjectives to school‐aged children with (D)LD. The focus on verbs and adjectives was motivated by evidence that these word types are more difficult to acquire than concrete nouns, particularly for children with (D)LD (Davies et al. 2023; Kan and Windsor 2010).

The implicit condition was the *reference condition*. The experimental conditions were: *+ explicit teaching*, *+ sign* and *+ explicit & sign*. Progress was assessed via two tasks: a word definition task (our primary outcome measure) and a sentence generation task (secondary outcome measure). We aimed to answer the following research questions:

1. Do participants make more progress on items in the experimental conditions, compared to items in the *reference condition*?
2. Is there an additional benefit from the combined *+ explicit & sign* condition, compared to the other two experimental conditions?

## 2 Methods

### 2.1 Study Design

The study employed a within‐subjects experimental design with randomisation and blinded outcome assessment. Each participant received vocabulary instruction under four different conditions: (1) the *reference condition* (implicit exposure only); (2) reference *+ explicit teaching*; (3) reference *+ sign* and (4) reference *+ explicit & sign*. There were 48 target words, with 12 words randomly allocated to each condition; six verbs and six adjectives. Two counter‐balanced word lists were created, such that words assigned to a signing condition for half of the participants were presented without signs to the other half of the cohort and vice versa. Words were further subdivided into story sets, with four target words presented within each story (see Appendix A for details of Word Allocation Lists 1 and 2).

**FIGURE 1:**
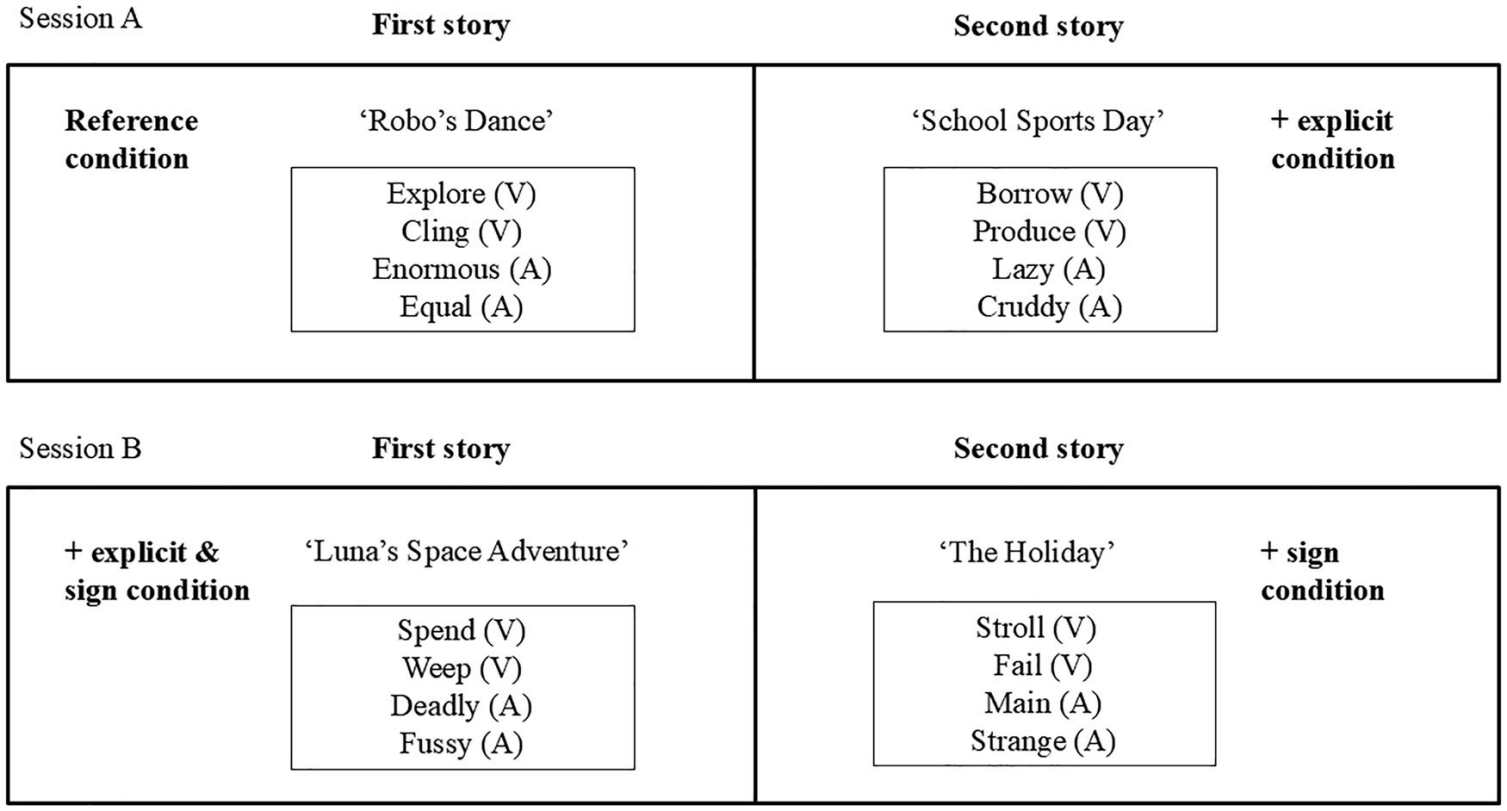
Starting week session contents for Child A, who was randomised to receive ‘Word Li 1’, beginning with the *reference* condition. Target words are shown in the inner rectangle. V, verb; A, adjective.

**FIGURE 2:**
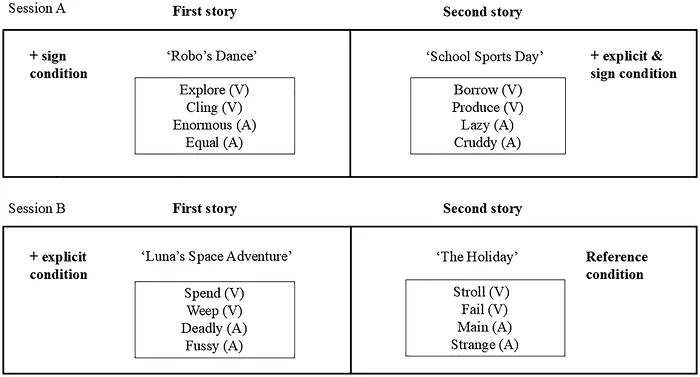
Starting week session contents for Child B, who was randomised to receive ‘Word Lis 2’, starting with *+ explicit & sign* condition. Target words are shown in the inner rectangle. V, verb; A, adjective.

All children worked on words across the four conditions, with the order of presentation randomised for each individual. Children moved through stories at different paces, depending on their response to probes at the start of each session, such that half of the children (*N* = 9) worked on all 48 words and others worked on fewer. The lowest number of words targeted was 32 (*N* = 4 children) and the mean was 42.22 words. Example pathways through intervention are provided for two children to illustrate the structure of sessions and how words were distributed across the conditions. Figures 1 and 2 show the starting week session content for Child A and Child B. Appendix B summarises the words and stories covered in subsequent sessions for each child.

Table 1 illustrates the study design and potential active ingredients within each condition, with some including multiple components, as described in Section 8.2.2 below.

**TABLE 1:** Study design and potential active ingredients within each condition.

Condition	Implicit intervention	Explicit teaching	Sign
Reference	✓	X	X
+ explicit	✓	✓	X
+ sign	✓	X	✓
+ explicit + sign	✓	✓	✓

#### 2.1.1 Ethics

This study was granted ethical approval by the Moor House Ethics Forum (reference number: 2023/6).

### 2.2 Participants

The study was carried out in a specialist school for children with language disorders in the United Kingdom. The intervention was primarily designed for Key Stage 2 students (aged 7–11 years) and target words and materials were selected with this age group in mind. However, speech and language therapists (SLTs) working across Key Stages 2–4 were invited to put forward students for the project, based on their professional judgement of whether each student may benefit from a story‐based vocabulary intervention.

Nineteen students completed pre‐intervention testing on our primary outcome measure (see below), five of whom were aged 12 years or above. Students met the inclusion criteria for the study if they scored <50% on the student‐generated part of the definitions task. Only one student was excluded from the study based on this criterion.

Eighteen students remained in the study (aged 7;09–15;02 years). All completed the full intervention protocol and follow‐up testing. Fourteen participants met diagnostic criteria for DLD, while four had language disorder associated with a biomedical condition (three with autism and one with a genetic disorder). Table 2 summarises participants’ ages and background language profiles.

**TABLE 2:** Participant characteristics.

	Age in years; months	Core language	Receptive language	Expressive language	Language content
Group mean	11;03	67.61	69.61	65.22	70.72
SD	1;11	13.16	12.37	12.40	11.21

*Note*: All language values are standard scores, derived from Wiig et al. (2017).

A side effect of our recruitment process was that older students who met criteria for inclusion had lower standardised language scores than their younger counterparts.

A Pearson correlation coefficient was computed to assess the linear relationship between age in months and Core Language standard scores from the Clinical Evaluation of Language Fundamentals, Fifth Edition (Wiig et al. 2017). There was a significant negative correlation, *r*(17) = −0.73, *p* = <0.001.

### 2.3 Intervention

One‐to‐one intervention was planned to take place twice a week for 30 min and to continue for 8 weeks (16 sessions, 8 h).

Two stories, each containing four target words, were presented in each session. During one session per week, all target words were signed (with or without the additional *+ explicit* story context). The other session in the week did not include signing (either in the *reference* or *+ explicit* story context). The starting order of sessions was randomised between participants, then alternated between conditions as illustrated in Figures 1 and 2 and Appendix B.

Intervention was delivered by participants’ usual SLT or an SLT familiar to them from within the school, as part of their typical speech and language therapy provision. Sessions were conducted in a quiet room on the school site. Six SLTs were involved in delivering the intervention, one of whom was the first author, who led the design of the intervention. The five other SLTs received training from the first author on how to deliver the research intervention. This training involved a 1‐h group meeting with all delivering SLTs to explain and demonstrate the intervention. All SLTs were provided with a written intervention protocol (see Appendix C), session plans and record sheets (Appendix D), access to video recordings for each story, scripted student‐friendly definitions and example sentences for the *+ explicit*, or *+ explicit & sign* conditions, and video recordings of signs for words targeted in the *+ sign* and *+ explicit & sign* conditions.

The SLTs worked together in the school, so any queries or concerns could be discussed and resolved quickly. Teaching of target words and signs for the project participants only took place in research intervention sessions, with typical classroom instruction and additional typical SLT intervention (group and in‐class support) focusing on other aspects of language.

#### 2.3.1 Target word selection

Our study targeted understanding and use of verbs and adjectives to support the development of ‘Tier Two’ vocabulary; words that are commonly used across academic and story contexts, but are not typically used in everyday conversation (Beck et al. 2013). Twenty‐four verbs and 24 adjectives were selected as intervention targets. Words were chosen with reference to Kuperman et al. (2012), in consultation with SLTs, and were matched across conditions for age of acquisition ratings (see Appendix E).

#### 2.3.2 Intervention Materials

Following target word selection, items were randomly assigned to four word‐learning conditions, then split into three story sets per condition (four words per story set, 12 words per condition). The first author created two child‐friendly stories per set (24 stories in total), using ChatGPT (OpenAI 2025a) to help generate ideas for characters, settings and age‐appropriate sentence contexts. A single image was generated to capture the theme of each story, using the DALL‐E 3 image generation model (OpenAI 2025b). Examples of stimulus stories and accompanying images are provided in Appendix F.

The third author, who is a Highly Specialist SLT and accredited Makaton^1^ and Signed English tutor, video‐recorded herself reading each story (and in the *+ sign* conditions, signing the target words). The SLT was visible in all videos with the accompanying image displayed in Microsoft PowerPoint. Two versions of each story were recorded: one without signing and another with signing of target words. This was to allow story sets to be counter‐balanced across participants, such that half of the participants watched a particular story with signing of target words, whilst the other half watched the same story without signing.

#### 2.3.3 Method and Order of Instruction

The experimental intervention was devised by the project team and was informed by previous word‐learning studies (Nash and Donaldson 2005; Storkel et al. 2017, 2019). The intervention protocol is presented in Supporting Information 1, described using the Template for Intervention Description and Replication (TiDier) framework (Hoffmann et al. 2014). Example signs are provided in Supporting Information 2.

**FIGURE 3:**
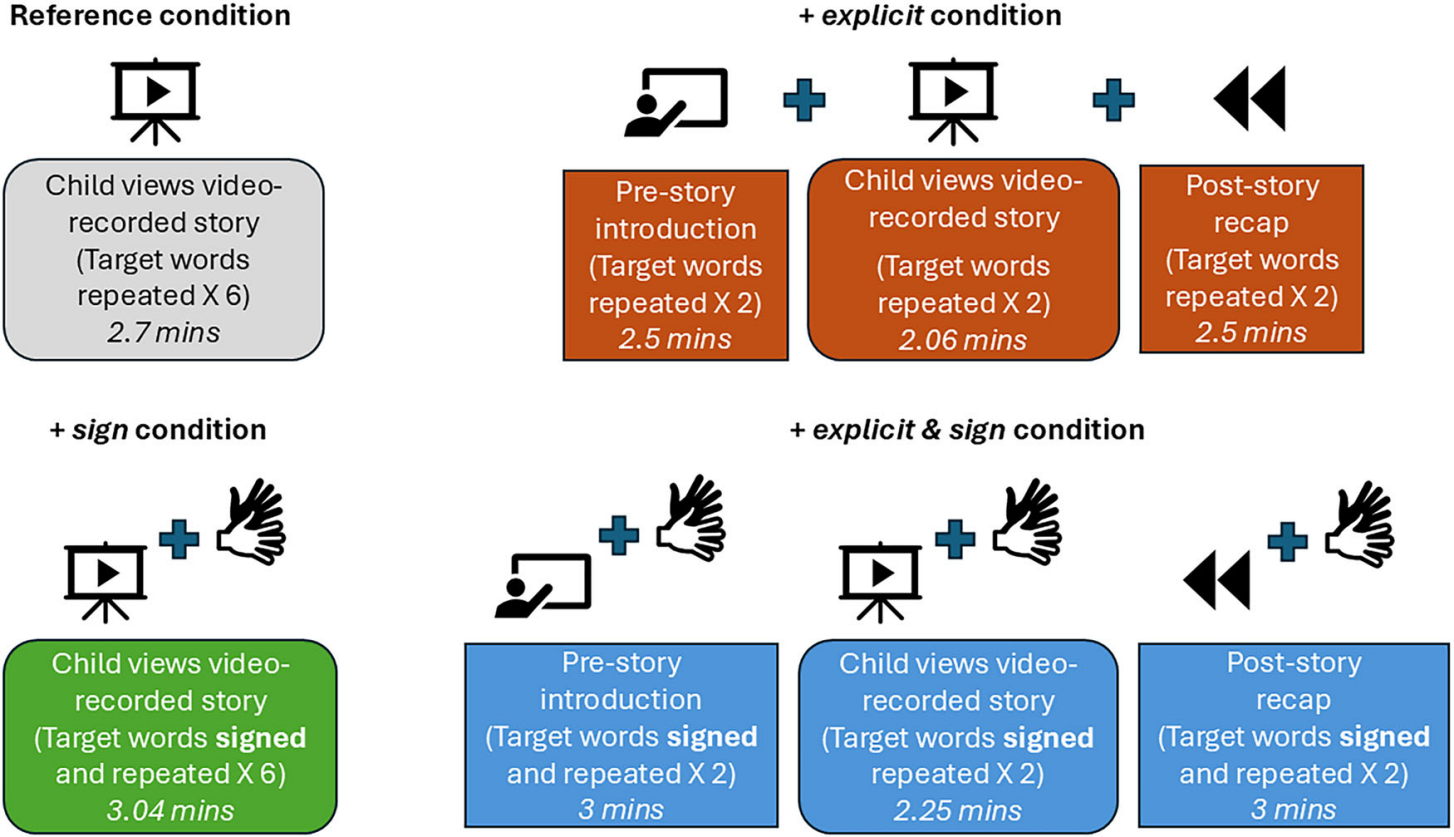
Intervention components for each condition.

In the initial session, the delivering SLT explained the intervention rationale in child‐friendly terms (e.g., to support understanding and remembering of new words, listening skills and understanding of stories). If the child was assigned to a *+ sign* condition for the first session, the therapist also explained that they would sometimes be using signs to support the child's learning of new words. Figure 3 summarises the key qualitative and quantitative components which were included in each condition. These are explained further below.

1. *Reference condition*: In this condition, participants watched and listened to a video‐recorded story. Participants were asked by the delivering SLT to ‘listen out for any new words in the story and see if you can work out what they mean’. Each of the four target words (two verbs and two adjectives) were repeated six times in different sentence contexts within the story. No further instruction on target word meanings was provided in the reference condition.
2. *+ explicit condition*: In this experimental condition, target words were introduced, and their meanings explicitly discussed prior to the corresponding video‐recorded story being played. The introduction included:
  a. Explicit description of the word class (with reference to terminology from the SHAPE CODING system^2^).
  b. Provision of a child‐friendly definition.
  c. Provision of a context sentence containing the target word and explanation of sentence meaning.
  d. Request for the child to retrieve the word.
  e. If the child could not remember the word, the following cueing hierarchy was used:
    i. Offer the child a phonemic cue.
    ii. SLT said the word and asked them to repeat it back.

Once the introductory activity was completed (with the SLT repeating each target word twice and the student once), the video‐recorded story was played. Target words were presented only twice each within this story. Following this, words were recapped following the same structure as the introductory section. In total, the child heard each target word six times per session to align with the number of presentations in the reference condition.

3. *+ sign condition*: This condition was the same as the reference condition, but with signs added each time a target word was spoken during the video presentation of stories. Participants were asked to ‘listen out for any new words in the story and watch the signs to see if you can work out what the words mean’.
4. *+ explicit & sign condition*: The final experimental condition combined active ingredients from all three previous conditions. Procedures followed those for the *+ explicit* condition, with the following additional components:
  a. In the pre‐story introduction, target words were introduced along with their accompanying signs.
  b. Participants were asked to copy the sign immediately after the SLT's demonstration.
  c. There was a request for them to retrieve signs, as well as word forms, both before and after the video viewing.
  d. Signs were incorporated in the video, as with the *+ sign* condition.
  e. The cueing hierarchy included two additional steps at the start:
    i. Ask them if they can show the sign.
    ii. If the student is unable to do this, the SLT provides the sign.

For all conditions, after the initial session, all subsequent sessions began with a quickfire recap of words covered in the previous session. During this recap, participants were asked to (a) generate a sentence and (b) give a definition of each target word. The child's response to this determined whether the same story would be repeated the following week again, as follows:

- If the participant scored 4/4 on the target words within either of the story sets which were taught in the previous session, they were judged ready to move on to a new set of target words from the same intervention condition in the next week's session.
- If the participant did not achieve mastery of all four target words after two sessions, the SLT changed from Story (a) to Story (b), containing the same target words.
- After four sessions, SLTs moved onto the next target word set within the same condition, irrespective of whether the child had achieved mastery or not. This was to counter fatigue and reduced motivation, based on feedback from piloting.

Following this procedure, the minimum number of repetitions for each word was six, and the maximum was 24.

### 2.4 Intervention Dosage, Timing and Spacing

Based on their progress through story sets, children were taught an average of 42.22 out of 48 words across all intervention conditions (mean of 10.56 per condition). Friedman chi‐squared test results indicated no significant difference between the numbers of words taught in each condition (see Table 3). While, on average, children required more exposures per word in the reference condition, compared to the three experimental conditions, in order to pass their next‐session probes for the given story set, this difference did not reach statistical significance (see Table 3).

**TABLE 3:** Summary of number of words taught and average repetitions per word in each intervention condition.

	Total words taught		Repetitions per word	
Condition	Mean	SD	Mean	SD
Reference	10.22	2.05	18.50	7.57
+ explicit	10.89	1.84	15.89	6.94
+ sign	10.67	1.94	17.89	5.95
+ explicit & sign	10.44	2.01	18.11	10.04
*p* value	*p* = 0.28		*p* = 0.30	

Table 4 indicates the timing and spacing of word presentations within the videos. This shows significant differences across conditions: videos with sign were, on average, longer than those without sign, with a slower overall rate of speech. However, words presented in the *+ explicit* and combined conditions involved greater spacing within sessions, due to exposures being distributed across the pre‐story introduction, story and post‐story recap. As individual sessions were not recorded, an estimate was made by therapists of the average time taken for the introduction and recaps: approximately 2.5 min for the *+ explicit* condition and 3 min for the *+ explicit & sign* condition. Variation was due to the child's response to intervention activities and the level of prompting required for them to retrieve each target word. Overall, the average time taken to present words in the *reference* condition was 2.68 min, compared to 3.04 min in the *+ sign* condition, 7.06 for the *+ explicit* and 8.25 for the *+ explicit & sign* condition (see Figure 3).

**TABLE 4:** Timing of word presentations within videos.

Condition	Length of videos (in minutes)		Rate of speech in video (overall words per min)	
	Mean	SD	Mean	SD
Reference	2.68	0.45	146.73	12.73
+ explicit	2.06	0.42	140.50	13.94
+ sign	3.04	0.35	129.10	7.85
+ explicit & sign	2.25	0.33	128.55	4.74
*p* value	*p* = <0.001		*p* = <0.01	

### 2.5 Intervention Fidelity

To support fidelity, all six delivering SLTs were given an intervention file, including scripts for introducing tasks, written definitions of target words, and the cueing hierarchy. Attendance and intervention fidelity were recorded through use of an intervention record sheet, which was completed by the delivering SLTs at the end of each session (see Appendix D). SLTs were asked to report any deviation from the session plan. When participants were unable to attend a session, SLTs continued by delivering the intervention plan from the missed session in the next session. Participants attended an average of 14 of the planned 16 intervention sessions (range: 12–16). Reduced attendance rates were due to participant or SLT illness, school trips or (in one case) the participant completing all target word sets and achieving 100% accuracy on within‐session probes before the end of the intervention period.

### 2.6 Outcome Measures

Participants were assessed on two separate tasks before and after the intervention. All assessments were carried out in an individual setting by qualified SLTs who were blind to the allocation of words to intervention conditions.

#### 2.6.1 Definitions Task

This was our primary outcome measure. The task had two parts. First, participants were presented with a target word and asked to ‘say what it means, in your own words’. For example, for the practice item ‘happy’, a correct response would be ‘feeling good’. Words were presented in a fixed, randomised order both verbally and in written form on a PowerPoint presentation, and participants were asked: ‘*Can you tell me what this word means?*’ Responses were transcribed live and definitions were subsequently scored as correct or incorrect according to a predefined list of acceptable definitions, which was based on piloting with a smaller group of students (*N* = 7; see Appendix G). Regardless of the accuracy of their response, participants were then presented with a second screen showing the word with three possible definitions (e.g., for the practice item *happy*, the alternatives were: ‘*feeling bad*’; ‘*feeling sad*’ or ‘*feeling good*’), and were asked to choose: ‘*Which one of these means happy?*’ Responses were categorized according to the following ordinal scale: score of 3 for a correct participant‐generated definition AND correct definition matching (from the three alternatives), score of 2 for a correct student‐generated definition, but incorrect definition matching, score of 1 for a correct definition matching, but incorrect participant‐generated definition, and a score of 0 for an incorrect or no response on both parts of the task.

#### 2.6.2 Sentence Generation Task

In this task, participants were given a target word and asked to: *‘Make up a sentence with the word in’*. They were given the following example and practice item: ‘If the word was *“laugh”*, I might say: *“The children laugh at the teacher”*. It doesn't need to be a long sentence, but it needs to make sense. Let's practise: Can you put this word in a sentence: *happy*?’ Responses were categorised according to the following ordinal scale: score of 2 for a full and complete sentence, which demonstrates clearly the participant's understanding of the target word, for example, ‘I *explore* the jungle’, score of 1 for a sentence which contains minor errors of semantics or grammar, or which uses a correct sentence frame but does not demonstrate clearly the student's understanding of the target word, e.g. ‘*I explore*’, and a score of 0 for an incorrect or no response.

### 2.7 Inter‐Rater Reliability

To evaluate whether the researcher‐generated tasks had adequate inter‐rater reliability (IRR), 20% of participant responses were re‐scored independently by the fifth author after the project's completion using the same list of acceptable and incorrect responses available to the original scorer. IRR for the student definitions task (where definitions were judged as either correct or incorrect) was assessed using Cohen's kappa (Hallgren 2012) to establish the degree to which the two independent coders provided consistency in their ratings of participants’ answers. For the sentence generation task (where student responses were rated as 0, 1 or 2), a weighted Cohen's kappa was used. This assigns partial credit for disagreements that are close in order, for example, if two raters Score 1 versus 2, this reflects a smaller degree of disagreement than a variation of 0 versus 2.

The resulting IRR for both tasks was in the strong agreement range (McHugh 2012); Cohen's kappa (κ) for participant definition = 0.83 (95% CI = 0.77–0.88); sentence generation = 0.86 (95% CI = 0.82–0.90).

### 2.8 Data Analysis

The two dependent variables resulting from the definition and sentence generation tasks were analysed with four multilevel models (one per task per research question) in a Bayesian framework, using Stan via the *brms* package in R (Bürkner 2021; Team 2025). A Bayesian statistical approach was chosen for three reasons. First, this prevents convergence issues often encountered in a frequentist framework especially given the relatively low sample size of the present study. Second, it provides direct probabilities of an effect so they can be readily interpreted in light of the study question. Third, Bayesian approaches have been argued to lead to fewer prediction errors than mixed effects models fitted with lme4 (Eager 2017).

The models used a logit‐link function, while assuming an ordinal distribution given that our two outcome measures were scales of 0,1,2,3 for the definition task and of 0,1,2 for the sentence generation task. Two fixed effects were considered: timepoint (pre‐ and post‐intervention) and condition (reference, *+ explicit teaching*, *+ sign*, *+ explicit & sign*) as well as their interaction. Both fixed effects were treatment coded, with the respective baseline reference levels being pre‐intervention and reference condition.

In Section 3, we discuss the estimates linked with the interactions between conditions and timepoint. These are estimates of how the post‐intervention scores for a given condition differ from what the model would have predicted given the difference in scores between pre‐ and post‐intervention in the reference condition and given the difference in pre‐intervention scores between any given experimental condition and the reference condition. Three random effects were also added to the models: random intercepts for each target word and for word class (adjective or verb) and random intercepts and slopes for participants following recommendations from Barr et al. (2013). This allows the models to control for pre‐intervention and learning rate differences between participants when estimating the effects associated with a given condition.

The detailed results of our models are shown in Appendix H, Table H1, where we report the models’ estimates and their probability of being positive, *P*(*β*) > 0. In a Bayesian framework, these values can be interpreted as the predicted increase, on the latent scale of the outcome measure, linked with a given factor, and the probability that the factor has a positive effect on the outcome measure. In order to facilitate the comparison between our results and that of other studies, we also calculated, from the model's predictions, the odds of improvement and their credible intervals following each parameter considered by the model and the probability of this, *P*(OR) > 1. These values provide an estimate of the size of the effect as the odds ratios are a direct indication of the odds of going up one category of the outcome scale while the *P*(OR) > 1 provides the probability that, for a given parameter, the odds are in favour of a higher outcome relative to the relevant reference.

More information about the models is included in the R analysis scripts available at this link: https://osf.io/tyeb2/overview?view_only=aa8b80876ee34a1dacbc0fc9001b2363.

## 3 Results

In this section, we report on the two Research Questions, first using the primary outcome measure (the definitions task) and then the sentence generation task. We also report on an exploratory analysis of the potential effect of age and background language scores.

### 3.1 Research Question 1

Do participants make more progress on items in the experimental conditions, compared to items in the reference condition?

#### 3.1.1 Definitions Task (Primary Outcome Measure)

**FIGURE 4:**
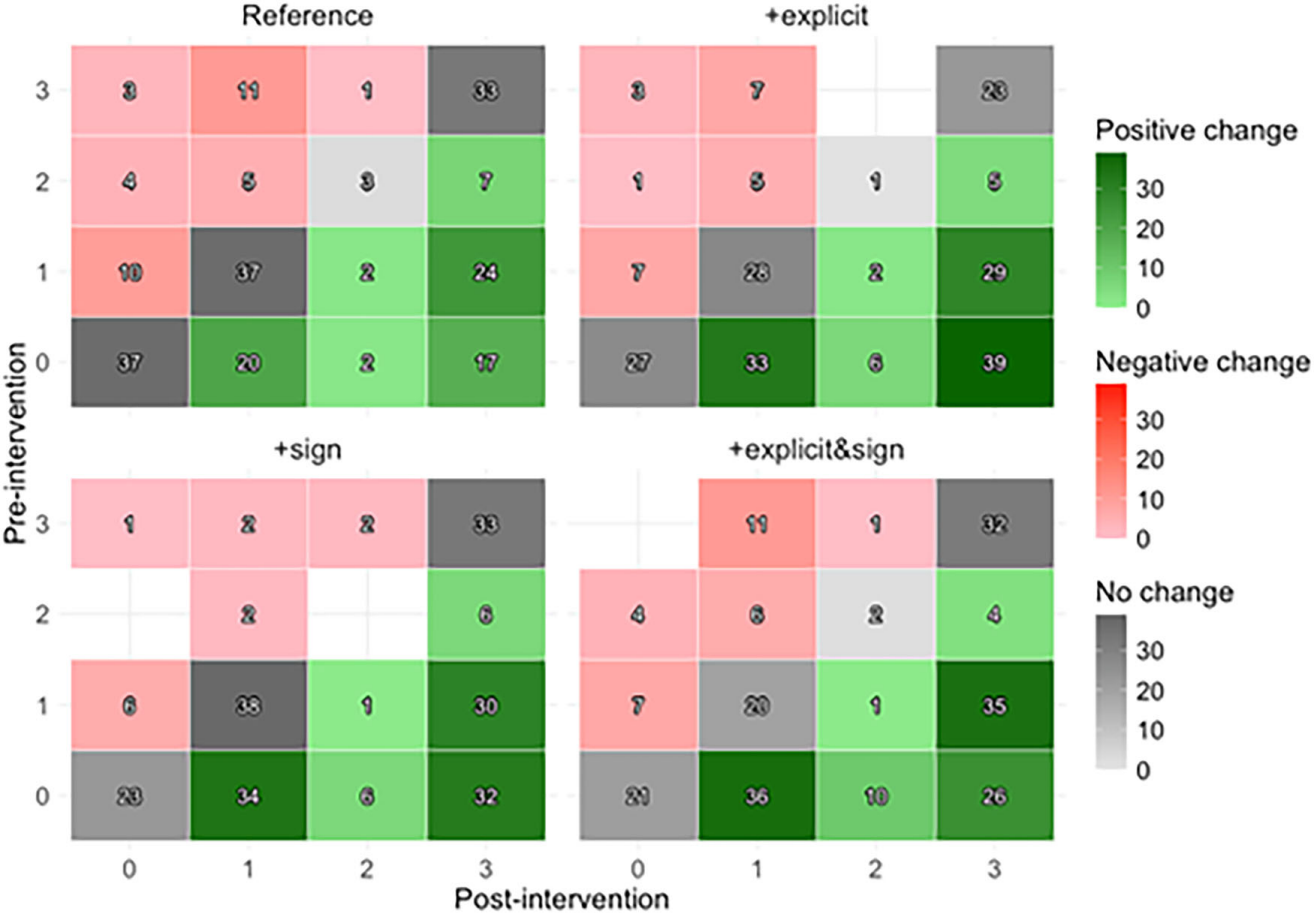
Heat‐map of the pre‐ and post‐intervention scores on the word definition task by condition. The numbers in the tiles are the number of words taught that fall in each category. Tiles in green represent a positive change (lower scores pre‐intervention than post‐intervention), tiles in grey are instances where there is no difference between pre‐ and post‐intervention scores and tiles in red indicate a negative change (post‐intervention scores are lower than pre‐intervention).

For each condition, the counts of words falling in each possible pre‐ and post‐intervention score combination are represented in Figure 4. Green tiles show a positive change while red tiles show a negative change. For both colours, the darker the tile, the more words fall into that category. Across all four conditions (including the reference condition), participants showed progress on definitions as indicated by higher numbers in the green tiles relative to the red tiles. However, the green tiles appear darkest in the three experimental conditions, which shows that more progress was made in these conditions relative to the reference.

**FIGURE 5:**
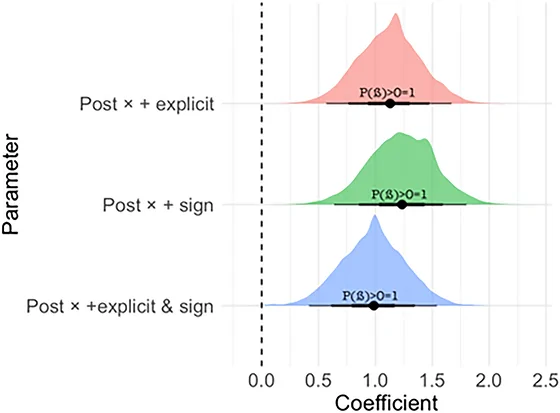
Posterior distribution of the coefficient estimates for the added post‐intervention effect of each experimental condition relative to the reference. The probability (*P*) indicated on each distribution corresponds to the proportion of the distribution that is above 0, which in turn indicates the probability that a given parameter is linked with an increase in outcome.

The trend visible in Figure 4 was confirmed statistically. A summary of the results is presented in Figure 5, and the detailed results of our models are shown in Appendix H, Table H1, where we report the models’ estimates and their probability of being positive, *P*(*β*) > 0 (the latter is also shown in Figure 5). Figure 5 shows the distribution of the coefficient estimates for the interaction between timepoint and condition. This can be interpreted as the progress that the model expects an experimental condition to yield in addition to the progress linked with the reference intervention and given the difference in pre‐intervention levels between the experimental condition and the reference intervention. Positive values (anything to the right of the dashed line) indicate that the parameter is linked with an increase in the outcome variable. In Figure 5, the whole of each of the three posterior distributions plotted is to the right of the dashed line, which shows that the corresponding parameters are always linked with an increase in definition task scores.

The statistical models revealed that the reference intervention led to post‐intervention vocabulary improvements (with a probability of 99%), but participants were predicted to have a 100% chance of greater progress on the words taught in the three experimental conditions than on those taught in the reference condition.

#### 3.1.2 Sentence Generation Task

**FIGURE 6:**
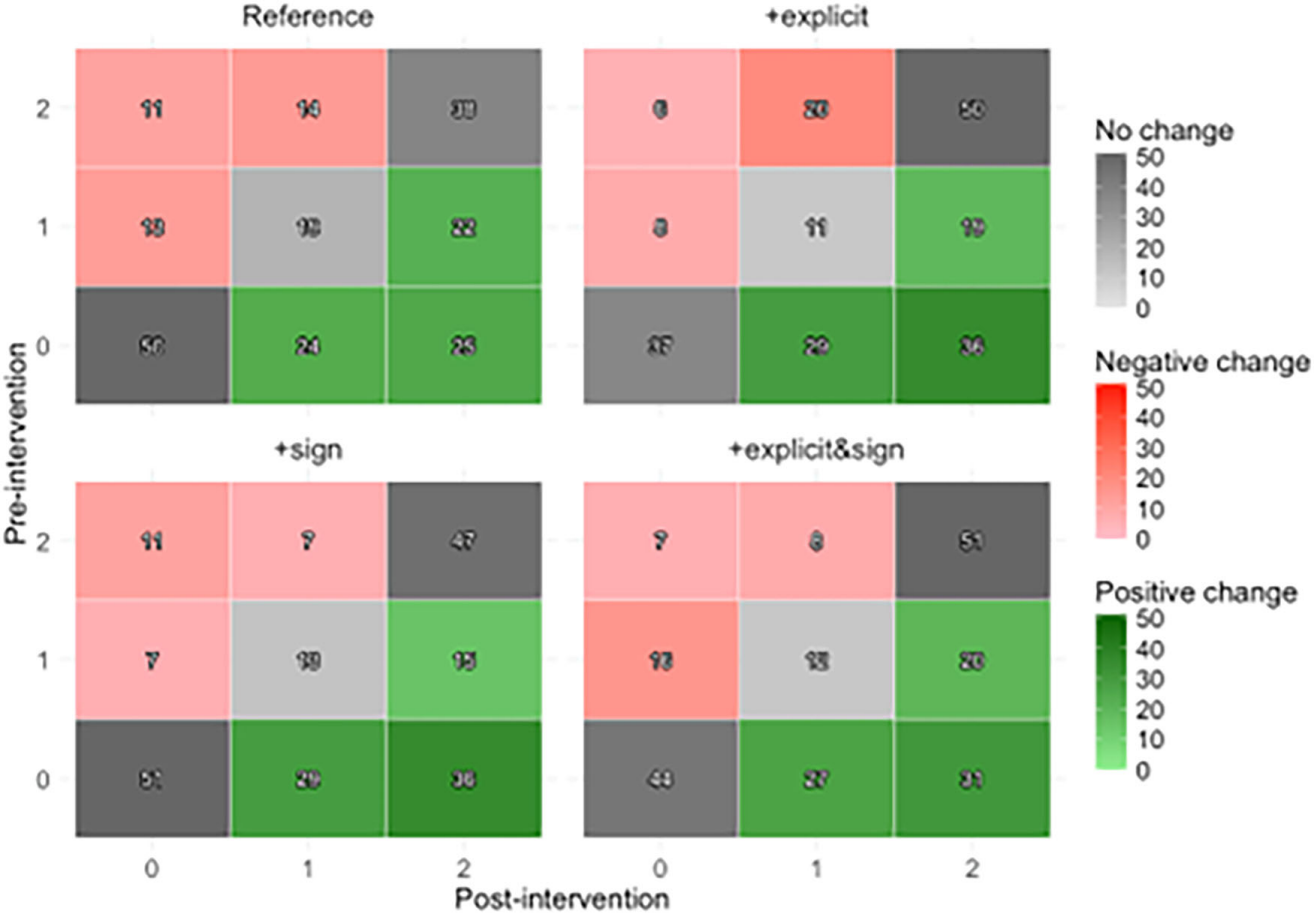
Heat‐map of the pre‐ and post‐intervention scores on the sentence generation task by condition (*reference, +explicit, +sign, +explicit & sign*). The numbers in the tiles are the number of words taught that fall in each category. Tiles in green represent a positive change (lower scores pre‐intervention than post‐intervention), tiles in grey are instances where there is no difference between pre‐ and post‐intervention scores and tiles in red indicate a negative change (post‐intervention scores are lower than pre‐intervention).

As for the definitions task, participants showed progress following intervention across all four conditions, including the reference condition. This can be seen by the higher numbers in the green tiles relative to the red tiles (i.e., there were more words on which participants had a better score post‐intervention relative to pre‐intervention than the other way around) in Figure 6. The comparison between intervention types cannot accurately be inferred from the count data directly. However, once again, the green tiles in the reference intervention are lighter in colour (i.e., contain fewer words) than in the other three conditions.

**FIGURE 7:**
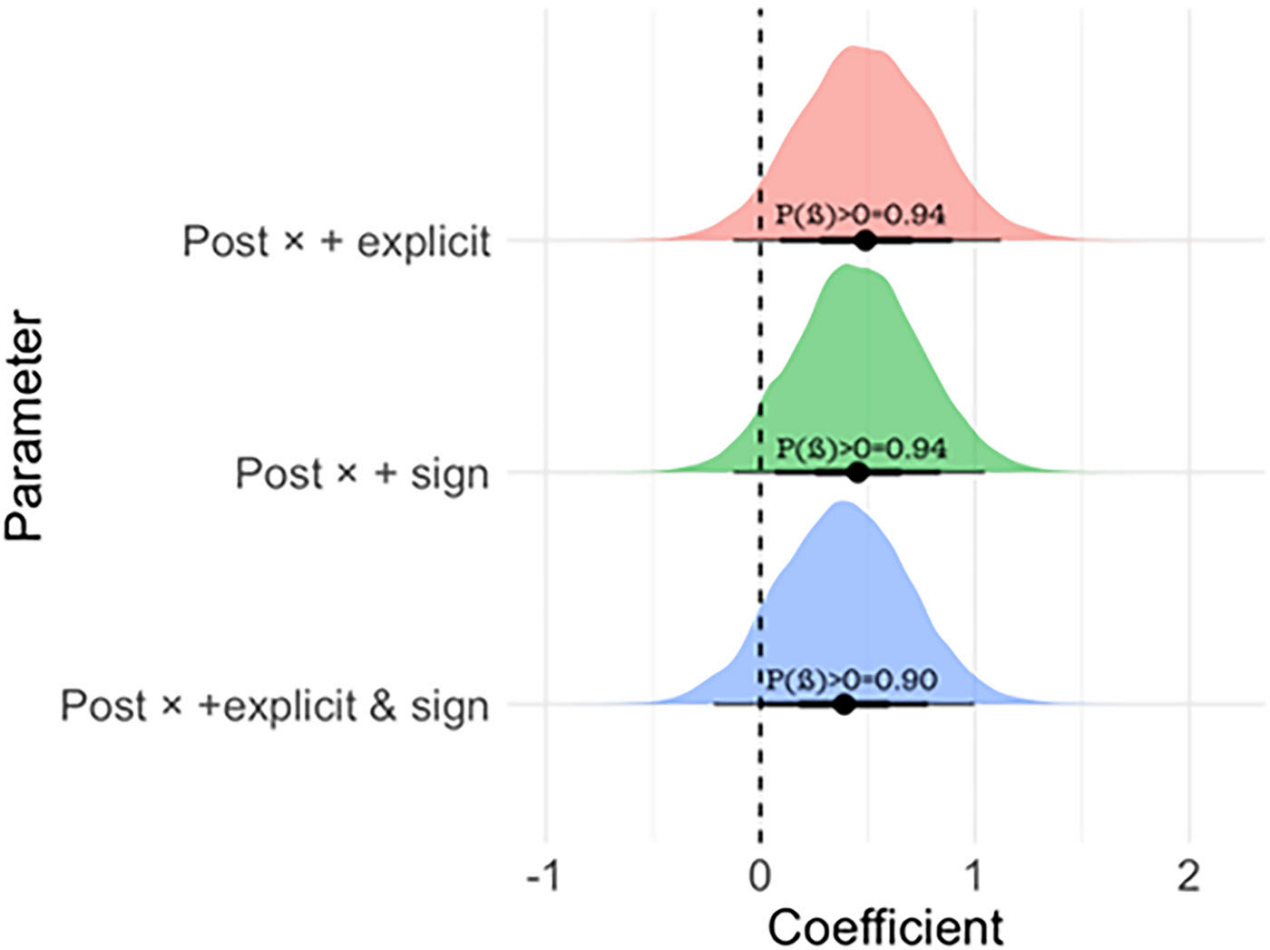
Posterior distribution of the coefficient estimates for the added post‐intervention effect of each experimental condition relative to the reference. The probability (*P*) indicated on each distribution corresponds to the proportion of the distribution that is above 0 which in turn indicates the probability that a given parameter is linked with an increase in outcome.

The results of the statistical models are shown in Figure 7 and Appendix H, Table H2. These revealed that the reference intervention led to post‐intervention vocabulary improvements in the sentence generation task (with 98% probability). The three experimental conditions again led to greater progress than the reference intervention, with probabilities ranging from 90% to 94% as illustrated by a small part of the distribution crossing the dashed line.

### 3.2 Research Question 2

Is there an additional benefit from the combined *+ explicit & sign* condition, compared to the other experimental conditions?

To answer Research Question 2, we removed the reference condition and fitted the data with the same models as for Research Question 1. We used the *+ explicit & sign* condition as reference in order to evaluate whether the *+ explicit* and the *+ sign* conditions were linked with less post‐intervention progress than the *+ explicit & sign* condition (i.e., there is an additional benefit of the combined condition, which might be expected). We therefore now report the probability that the estimate of the interaction between post‐intervention and the *+ explicit* or the *+ sign* condition is negative, *P*(*β*) < 0.

#### 3.2.1 Definition Task (Primary Outcome Measure)

**FIGURE 8:**
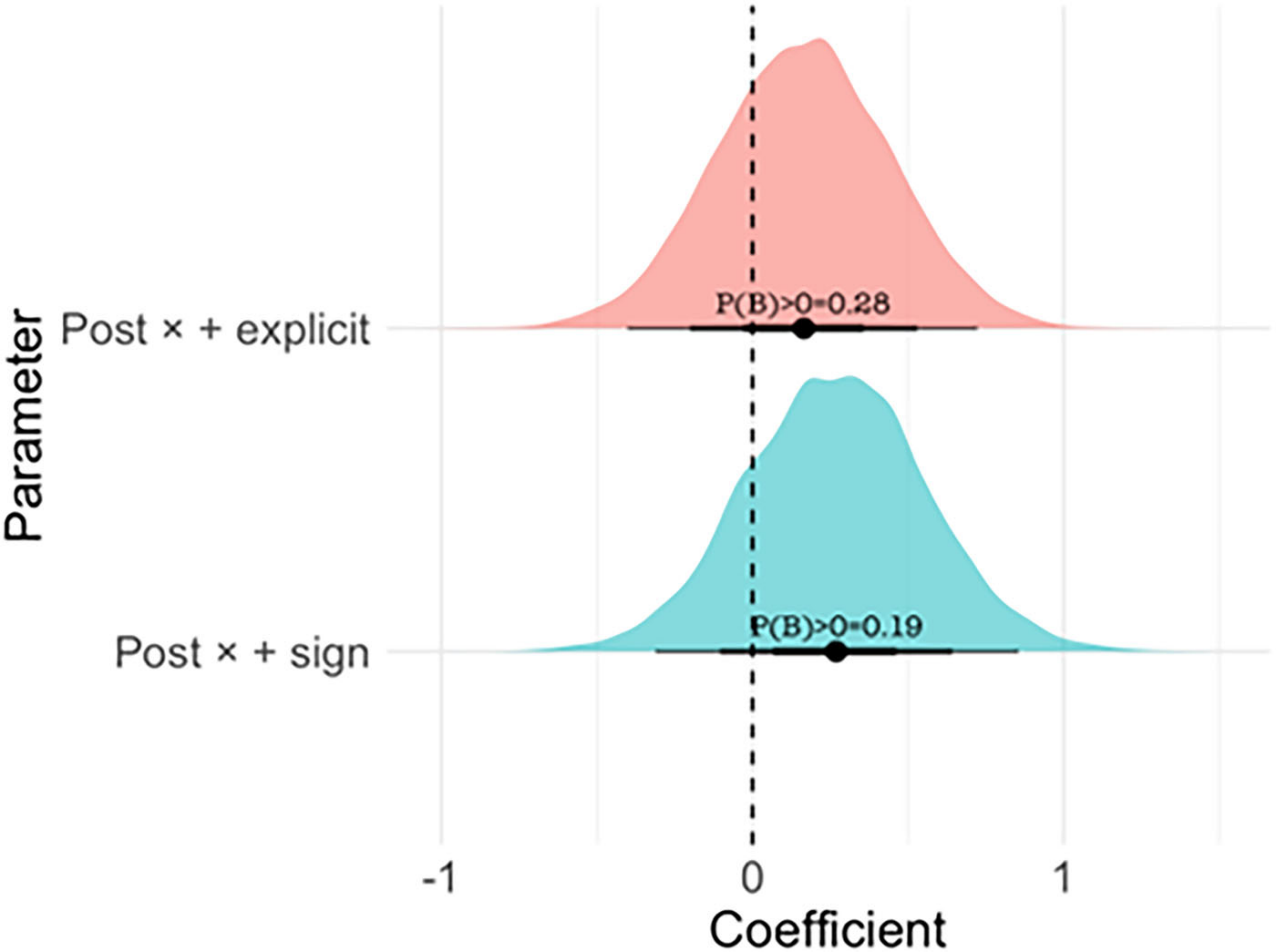
Definition task: posterior distribution of the coefficient estimates for the added post‐intervention effect of the *+explicit* and *+sign* condition relative to *+explicit & sign*. The probability (*P*) indicated on each distribution corresponds to the proportion of the distribution that is above 0 which in turn indicates the probability that a given parameter is linked with an increase in outcome.

The results of the statistical models are shown in Figure 8 and Appendix H, Table H3. These revealed that neither the *+ explicit* nor the *+ sign* condition led to *less* progress than the combined *+ explicit & sign* condition, so we can conclude that there is no additional benefit of the combined *+ explicit & sign* condition over the other two experimental conditions. Indeed, the bulk of the posterior distributions represented in Figure 8 are positive (to the right of the dashed line) indicating the opposite tendency, namely that the *+ explicit* and *+ sign* conditions are predicted to lead to greater progress than the combined *+ explicit & sign* condition. The part of the distribution below zero however is too large to firmly conclude that the *+ explicit & sign* condition led to less progress on the definition task than the other two conditions.

#### 3.2.2 Sentence Generation Task

**FIGURE 9:**
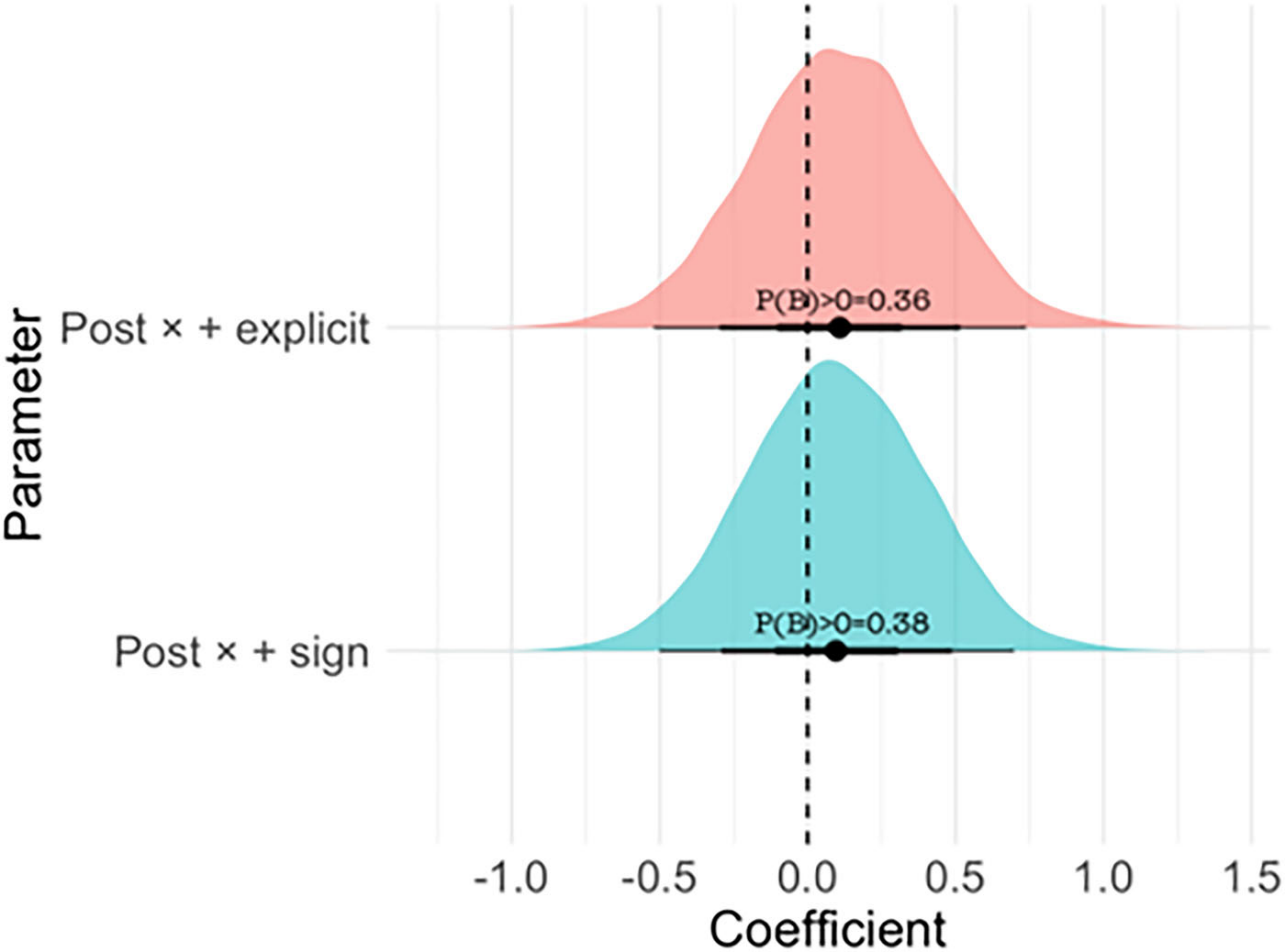
Sentence generation task: posterior distribution of the coefficient estimates for the added post‐intervention effect of the *+explicit* and *+sign* condition relative to *+explicit & +sign*. The probability indicated on each distribution corresponds to the proportion of the distribution that is above 0 which in turn indicates the probability that a given parameter is linked with an increase in outcome.

The results of the statistical models are shown in Figure 9 and Appendix H, Table H4. As for the definition task, the *+ explicit & sign* condition was not linked with better progress post‐intervention relative the other two experimental conditions.

### 3.3 Exploratory Analysis of Effect of Age and Language Scores

Given that we had a wide range of ages within our participant sample, and that age and standard language scores were highly correlated, we wanted to explore whether there was a relationship between age, Core Language standard scores and progress in response to each of the intervention conditions. We ran 16 (one per condition, per task for both age and language) Pearson correlations corrected for multiple comparison with Benjamini–Hochberg procedure. None of the correlations was significant, with values ranging from *r*(16) = –0.20 to *r*(16) = –0.47 and with the lowest *p* value being *p* = 0.13.

Given the confound between age and standard scores and the small sample size, we can, however, not draw any strong conclusions from this lack of association between standardised language scores and age on one side and progress following the four different interventions on the other.

## 4 Discussion

This small‐scale study has extended the use of story‐based vocabulary intervention to school‐aged students with (D)LD. As for previous research involving pre‐schoolers with and without language difficulties (Nash and Donaldson 2005), our participants showed additional gains from the inclusion of explicit teaching. Our study also adds to previous evidence that children with (D)LD can benefit from augmentative signing to support vocabulary learning (van Berkel‐van Hoof et al. 2019).

Repetitions and dosage were kept constant within sessions, across all conditions. However, children moved through story sets at different paces, such that half of the children (*N* = 9) were taught all 48 words and others were taught fewer. The lowest number of words targeted was 32 (*N* = 4 children) and the mean was 42.22 words. This total is higher than previous studies involving pre‐school children (e.g., Storkel et al. 2017, 2019), although the number of words taught per condition was comparable (mean of 10.56 in our study versus 12 for Storkel and colleagues) and the total words taught aligned with vocabulary studies involving school‐aged children (e.g., Best et al. 2021). There was no significant difference in the average number of words targeted across learning conditions

On average, participants progressed from scoring full marks for nine items on the primary outcome measure pre‐intervention to almost 21 items post‐intervention across all conditions, a mean increase of 11.78 words, as shown by count data for the definitions task (see Figure 3). This is well above the modest gains reported in previous studies involving younger children with language disorders, for example, McGregor et al. (2021), though the McGregor intervention was delivered within a group setting and focused on science‐related vocabulary which involved additional teaching to support children's understanding of underlying concepts. The number of words learned from our study were comparable to those reported in Best et al. (2021), which involved school‐aged children, employing elaboration of semantic and phonological features of target vocabulary using word webs. However, the Best et al. (2021) study focused on concrete nouns. In contrast, our intervention targeted verbs and adjectives, which are more abstract and difficult to acquire than concrete nouns, particularly for children with (D)LD.

Another important finding from our study is that children required an average of 17.6 exposures per word (across conditions) to achieve word learning gains—well below the 36 exposures reported by Storkel et al. (2017) for pre‐schoolers with DLD. It appears that our school‐aged children were able to make use of the rich and varied semantic information embedded within the story context and that this had benefits for their attention and engagement to support their learning and remembering of new words. This is in line with Roembke et al.’s (2023) account of stories supporting semantic processing and encouraging cross‐situational generalisation of vocabulary knowledge. Incorporating spoken stories into interventions for school‐aged children with (D)LD is a promising avenue, as it may help compensate for the literacy difficulties which often accompany language disorders. While typically‐developing teenagers often acquire new words via reading (Nagy and Herman 1987), those with (D)LD may continue to benefit from more oral input at a later stage of their word learning journeys.

Overall, participants showed similar benefits from adding the single component of signing or adding explicit teaching, which combined multiple intervention components, including modelling and retrieval. This is a striking finding, given the total differences in delivery time that were associated with our *+ sign* and *+ explicit* learning conditions (3.04 min, compared to 7.06 min per story set)—nearly 2.5 times longer in the *+ explicit* condition. The increased efficiency of signing would have important implications in the classroom, or when scaled up across a series of SLT intervention sessions. These differences in efficiency came despite the SLT's rate of speech being slower within the signing videos, a finding which aligns with Rozen‐Blay et al. (2022). It is likely that this slower speech rate was a consequence of dual‐task demands and cross‐modal coordination for the therapist, but may have carried the incidental benefit of supporting students’ processing of spoken, as well as visual input—something that was not controlled for directly within this study.

Other potential mechanisms of change for signing were increased attention (Wakefield et al. 2018) and the presentation of nuances of meaning that might not be present in the story itself (Novack and Goldin‐Meadow 2015). Though the degree of iconicity was not tested within this study, previous research indicates that signing has the most benefits for word learning when the signs used show a clear resemblance to their word referent (van Berkel‐van Hoof et al. 2016). This could be explored further in future studies, though some concepts, such as ‘manage’ and ‘main’, are evidently harder to represent visually than change‐of‐state verbs, such as ‘attach’.

Our study does not allow us to determine which of the explicit teaching elements are the most important active ingredients, or whether all are necessary to support vocabulary learning. Further research is necessary to explore this further, by adding or withholding potential active ingredients across different explicit word learning conditions.

Similar benefits were observed when explicit teaching and signing were combined (i.e., no additional benefit from their combination, with hints that this may even lead to poorer performance). This runs counter to the dual coding theory (Li et al. 2022), which holds that presenting information in two modalities can strengthen encoding and memory traces. Instead, these findings could be interpreted in light of the redundancy principle (Kalyuga and Sweller 2014), suggesting that coordinating information in different forms may have interfered with children's learning by increasing cognitive and working memory load.

The *+ explicit & sign* condition was the only learning context which included the child being required to produce the signs for target words themselves. Previous studies on embodiment have found that prompting children to gesture while learning a new concept can help them represent and store new ideas (Cook et al. 2008). It may be that each of the individual components (+ *sign* and + *explicit*) was already very effective, such that it was not possible to show additional benefits from integrating these supports. Alternatively, children in our study may have benefited more from these strategies being used sequentially, rather than concurrently, to ease processing demands.

Our findings combine to suggest that clinicians and educators should consider using either explicit teaching or signing alongside speech during everyday activities in the class or clinic room. The benefit of signing is that this can be integrated with classroom talk, with this approach taking less time to deliver than additional explicit teaching, because it happens in parallel with implicit exposure. In our study, participants showed benefits from observing signs whilst listening to a story, even when not asked to retrieve target signs or words themselves. Where practitioners are less confident in using signing or gestures to support word learning, they could choose instead to incorporate explicit teaching, such as additional explanations of words, offering examples of sentences where they appear and seeking regular retrieval.

Whilst our study did not identify any clear association between participants’ age or language profiles and their response to intervention, in practice the selection of support strategies should take into consideration the individual needs and preferences of the learner. For example, in our specialist setting, some students report enjoying and finding benefit from learning and using signs, whereas others (often older students) prefer to use other word learning aids, such as explicit definitions and/or orthographic support.

### 4.1 Limitations

This study was limited by the relatively small number of participants and by the pre‐post design. Ideally, word learning studies should incorporate multiple baseline testing, as well as follow‐up assessments to test retention of newly‐taught words (Howard et al. 2015). Retesting words after a longer time‐scale could help establish whether different active ingredients (e.g., explicit teaching and/or signing) can differentially affect how well vocabulary is retained in the longer term. Future studies could address these methodological limitations, whilst potentially including a control group to further enhance experimental rigour and evaluate the effectiveness of the reference intervention. Testing children on all words within each session would also enable analysis of how participants’ raw scores changed across the 8‐week intervention period, but this could adversely affect children's motivation and the time available for intervention.

A larger‐scale study could allow us to investigate potential small effects, for example, differences between the three experimental conditions and potential differences in dosage (the number of exposures required to learn words within each condition). A larger sample would also enable further exploration of whether participant characteristics such as age or language abilities may influence participants’ response to intervention.

### 4.2 Conclusions

This preliminary study highlights the need for systematic evaluation of the active ingredients within vocabulary interventions. Understanding not only the individual contribution of these components but also how they work together is essential, as simply adding more elements does not guarantee better progress. Our findings have practical implications for vocabulary instruction, both in SLT sessions and within the classroom, indicating that staff should prioritise incorporating either augmentative signing or explicit teaching strategies whenever feasible. Doing so can provide targeted support for students’ vocabulary development, with potential to enhance both oracy and literacy outcomes.

## Funding

The project was funded by Moor House School & College.

## Ethics Statement

This study was granted ethical approval by the Moor House Ethics Forum (reference number: 2023/6).

## Conflicts of Interest

All the authors are employed by Moor House School & College, where the study was carried out.

## Supporting information




**Supporting Information File 1**: jlcd70317‐supp‐0001‐SuppMat.docx

**Supporting Information File 2**: jlcd70317‐supp‐0002‐SuppMat.pptx

## Data Availability

Analysis scripts from this project are available at: https://osf.io/tyeb2/overview?view_only=aa8b80876ee34a1dacbc0fc9001b2363.

## Endnotes

1 Makaton is a communication system, which uses augmentative signing and symbols alongside speech to support understanding and expression for people with language and/or learning difficulties.

2 The SHAPE CODING system is a visual language support system that is used routinely by SLTs and classroom practitioners across our specialist school setting. For more details of this approach and research evidence to support this, see: www.shapecoding.com

## Appendix A

### Counter‐Balanced Word Allocation Lists and Story Sets

**Word List 1**

**Table:**

	Reference condition	+ Explicit condition	+ Sign condition	+ Explicit & sign condition
Story set 1	Explore (V)	Borrow (V)	Stroll (V)	Spend (V)
	Cling (V)	Produce (V)	Fail (V)	Weep (V)
	Enormous (A)	Lazy (A)	Main (A)	Deadly (A)
	Equal (A)	Cruddy (A)	Strange (A)	Fussy (A)
Story set 2	Fetch (V)	Organise (V)	React (V)	Attend (V)
	Dazzle (V)	Skedaddle (V)	Chuck (V)	Solve (V)
	Dreadful (A)	Homemade (A)	Single (A)	Immediate (A)
	Serious (A)	Wild (A)	Respectful (A)	Floppy (A)
Story set 3	Provide (V)	Deny (V)	Accomplish (V)	Manage (V)
	Participate (V)	Protect (V)	Attach (V)	Agree (V)
	Useful (A)	Wonderful (A)	Greasy (A)	Chilly (A)
	Toothless (A)	Heavenly (A)	Hilly (A)	Triple (A)

Abbreviations: V, verb; A, adjective.

**Word List 2**

**Table:**

	Reference condition	+ Explicit condition	+ Sign condition	+ Explicit & sign condition
Story set 1	Stroll (V)	Spend (V)	Explore (V)	Borrow (V)
	Fail (V)	Weep (V)	Cling (V)	Produce (V)
	Main (A)	Deadly (A)	Enormous (A)	Lazy (A)
	Strange (A)	Fussy (A)	Equal (A)	Cruddy (A)
Story set 2	React (V)	Attend (V)	Fetch (V)	Organise (V)
	Chuck (V)	Solve (V)	Dazzle (V)	Skedaddle (V)
	Single (A)	Immediate (A)	Dreadful (A)	Homemade (A)
	Respectful (A)	Floppy (A)	Serious (A)	Wild (A)
Story set 3	Accomplish (V)	Manage (V)	Provide (V)	Deny (V)
	Attach (V)	Agree (V)	Participate (V)	Protect (V)
	Greasy (A)	Chilly (A)	Useful (A)	Wonderful (A)
	Hilly (A)	Triple (A)	Toothless (A)	Heavenly (A)

Abbreviations: V, verb; A, adjective.

## Appendix B

### Example Pathways Through Intervention for Two Children

*N.b. Children moved through stories at different paces, depending on their response to probes at the start of each session. Order of presentation was varied across sessions to control for sequencing effects. Stories were changed after two consecutive sessions to maximise participant interest and engagement*.

Child A: Randomised to Receive ‘Word Set 1’, Beginning With *+ Sign* Story

**Table:**

Session	First story				Second story			
	Condition	Story set	Title	Target words	Condition	Story set	Title	Target words
1	*Reference*	1a	‘Robo's Dance’	Explore	*+ explicit*	1a	‘School Sports Day’	Borrow
				Cling				Produce
				Enormous				Lazy
				Equal				Cruddy
2	*+ explicit & sign*	1a	‘Luna's Space Adventure’	Spend	*+ sign*	1a	‘The Holiday’	Stroll
				Weep				Fail
				Deadly				Main
				Fussy				Strange
3	*+ explicit*	1a	‘School Sports Day’	Borrow	*Reference*	1a	‘Robo's Dance’	Explore
				Produce				Cling
				Lazy				Enormous
				Cruddy				Equal
4	*+ sign*	1a	‘The Holiday’	Stroll	*+ explicit*	1a	‘Luna's Space Adventure’	Spend
				Fail	*& sign*			Weep
				Main				Deadly
				Strange				Fussy
5	*Reference*	1b	‘Magic Washing Machine’	Explore	*+ explicit*	1b	‘Dinosaur Picnic’	Borrow
				Cling				Produce
				Enormous				Lazy
				Equal				Cruddy
6	*+ explicit & sign*	2a	‘Rosie's Big Day’	Attend	*+ sign*	1b	Sparky's Adventures	Stroll
				Solve				Fail
				Immediate				Main
				Floppy				Strange
7	*+ explicit*	1b	‘Dinosaur Picnic’	Borrow	*Reference*	1b	‘Magic Washing Machine’	Explore
				Produce				Cling
				Lazy				Enormous
				Cruddy				Equal
8	*+ sign*	1b	Sparky's Adventures	Stroll	*+ explicit & sign*	2a	‘Rosie's Big Day’	Attend
				Fail				Solve
				Main				Immediate
				Strange				Floppy
9	*Reference*	2a	‘Finlay's swimming lesson’	Fetch	*+ explicit*	2a	‘A Day at the Zoo’	Organise
				Dazzle				Skedaddle
				Dreadful				Homemade
				Serious				Wild
10	*+ explicit & sign*	2b	‘The School Play’	Attend	*+ sign*	2a	‘Caiden's Day at School’	React
				Solve				Chuck
				Immediate				Single
				Floppy				Respectful
11	*+ explicit*	2a	‘A Day at the Zoo’	Organise	*Reference*	2a	‘Finlay's swimming lesson’	Fetch
				Skedaddle				Dazzle
				Homemade				Dreadful
				Wild				Serious
12	*+ sign*	2a	‘Caiden's Day at School’	React	*+ explicit & sign*	2b	‘The School Play’	Attend
				Chuck				Solve
				Single				Immediate
				Respectful				Floppy
13	*Reference*	2b	‘The Shopping Trip’	Fetch	*+ explicit*	2b	‘A Forest Adventure’	Organise
				Dazzle				Skedaddle
				Dreadful				Homemade
				Serious				Wild
14	*+ explicit & sign*	3a	‘The Football Match’	Manage	*+ sign*	2b	‘School Trip’	React
				Agree				Chuck
				Chilly				Single
				Triple				Respectful
15	*+ explicit*	3a	‘The Royal Wedding’	Deny	*Reference*	3a	‘Timmy the Dragon’	Provide
				Protect				Participate
				Wonderful				Useful
				Heavenly				Toothless

*Note*: NB Child A was absent for one intervention session.

Child B: Randomised to Receive ‘Word Set 2’, Beginning With *+ Explicit & Sign* Story

**Table:**

Session	First story				Second story			
	Condition	Story set	Title	Target words	Condition	Story set	Title	Target words
1	*+ explicit & sign*	1a	‘School Sports Day’	Borrow	*+ sign*	1a	‘Robo's Dance’	Explore
				Produce				Cling
				Lazy				Enormous
				Cruddy				Equal
2	*Reference*	1a	‘The Holiday’	Stroll	*+ explicit*	1a	‘Luna's Space Adventure’	Spend
				Fail				Weep
				Main				Deadly
				Strange				Fussy
3	*+ sign*	1a	‘Robo's Dance’	Explore	*+ explicit & sign*	1a	‘School Sports Day’	Borrow
				Cling				Produce
				Enormous				Lazy
				Equal				Cruddy
4	*+ explicit*	2a	‘Rosie's Big Day’	Attend	*Reference*	2a	‘Caiden's Day at School’	React
				Solve				Chuck
				Immediate				Single
				Floppy				Respectful
5	*+ explicit & sign*	1b	‘Dinosaur picnic’	Borrow	*+ sign*	1b	‘The Magic Washing Machine’	Explore
				Produce				Cling
				Lazy				Enormous
				Cruddy				Equal
6	*Reference*	2a	‘Caiden's Day at School’	React	*+ explicit*	2a	‘Rosie's Big Day’	Attend
				Chuck				Solve
				Single				Immediate
				Respectful				Floppy
7	*+ sign*	2a	‘Finlay's Swimming Lesson’	Fetch	*+ explicit & sign*	2a	‘A Day at the Zoo’	Organise
				Dazzle				Skedaddle
				Dreadful				Homemade
				Serious				Wild
8	*+ explicit*	3a	‘The Football Match’	Manage	*Reference*	3a	‘Sammy Snail's Big Adventure’	Accomplish
				Agree				Attach
				Chilly				Greasy
				Triple				Hilly
9	*+ explicit & sign*	2a	‘A Day at the Zoo’	Organise	*+ sign*	2a	‘Finlay's Swimming Lesson’	Fetch
				Skedaddle				Dazzle
				Homemade				Dreadful
				Wild				Serious
10	*Reference*	n/a	FINISHED		*+ explicit*	n/a	FINISHED	
11	*+ sign*	2b	‘Shopping Trip’	Fetch	*+ explicit & sign*	3a	‘Royal Wedding’	Deny
				Dazzle				Protect
				Dreadful				Wonderful
				Serious				Heavenly
12	*+ explicit & sign*	3b	‘The Bear, the Wizard and the Bunny’	Deny	*+ sign*	3a	‘Timmy the Dragon’	Provide
				Protect				Participate
				Wonderful				Useful
				Heavenly				Toothless
13	*+ sign*	3a	‘Timmy the Dragon’	Provide	*+ explicit & sign*	n/a	FINISHED	
				Participate				
				Useful				
				Toothless				

*Note*: NB intervention ceased early due to partipant achieving 100% on within‐session probes.

## Appendix C

### Intervention Protocol for Story‐Based Vocabulary Intervention Programme

**Materials**:

- Sign videos (needed for Explicit Sign condition only)
- Implicit story videos
- Explicit story videos
- Written stories with intro and recaps for Explicit conditions

**Initial Session**:

*You will need*: *Access to the correct stories, images and* ***sign videos if child is assigned to the explicit signing condition for this session*** *. Session plan/record sheet*.

1. Explain therapy rationale in child friendly terms—this intervention will support
  ‐ Understanding of Words
  ‐ Remembering New Words
  ‐ Listening to and Understanding Stories

**If child is assigned to ‘with signing’ condition for this session, explain you and students will use sign to support learning. Bold instructions for ‘with signing’ condition only**.

*NB The therapy is designed so that SLT says/* ***signs*** *each target word 6 times per session. Please aim to stick to this so that we can control for number of exposures in the sign versus no sign conditions*.

2. First story
  ‐ Explicit condition
  - If the child is assigned to receive an ‘*explicit*’ story first in this session, introduce the target words as indicated on Story sheet 1a (**with sign if child has been assigned to receive this for this session). For the signing condition, you will be prompted to ask the child to do the sign**.
  - Watch the 1a story video with the child, encouraging them to listen out for the target words **and look at signs if they are in the signing conditions ‘as this will help them understand and learn the words’**.
  - Recap the words (**and signs**) and check which ones the child can remember, as indicated on the story sheet. *If the child cannot remember a word, we will use the following cueing hierarchy:* ***For signing condition only, ask them if they can do the sign. If not, SLT gives them the sign.*** *If they still can't remember the word, the next step will be offering a phonemic cue. If they still can't retrieve the target, then we say the word and ask them to repeat it back*
  ‐ Implicit condition:

If child is assigned to receive an ‘*implicit*’ story first, watch the 1a story video with the child, encouraging them to listen out for the new words and see if they can work out what they mean. **If the child has been assigned to the signing condition for this session, encourage them to watch the signs ‘as this will help them to understand and remember the words’**.

3. Second story

Follow the same procedures as for the first story, above. You will be using Story 1a from the same signing condition as above, but the opposite Explicit/Implicit condition.

4. Student self‐evaluation

Use departmental rating scale/add to database, if appropriate.

**Session Two Onwards**:

*You will need: Access to words taught in the previous session for quickfire recap. Story videos for today's word sets. Print outs of explicit story intro and recap for today's words. Session plan/record sheet. **Sign videos for explicit words if child is assigned to the signing condition for this session (as you will need to sign during the intro and recap)***.

1. Quickfire recap of words taught in the previous session. Go through each of the eight previously taught words and ask children to (a) put the word in a sentence and (b) tell you what the word means. Record the child's responses on the session notes sheet. This will determine whether you think the child is secure in their understanding of the word to help you decide which stories will be worked on for the NEXT session. It will **not** affect plans for the current session. See ‘deciding when to move on’, below.
2. First story
  If the child was assigned to the no sign condition in the previous session, then this session's words will all be **signed**, and vice versa.
  If the child started with *‘explicit’* condition last time, then begin with an ‘*implicit’* story today and vice versa. Start with Story 1a for the appropriate condition.
3. Second story
  The signing or no signing condition will be the same as for Story 1. If you began with an *‘explicit’* story, then Story 2 will be *‘implicit’* and vice versa. Start with Story 1a for the appropriate condition.

**How to know when to move onto a new story or stories**:

This is partly determined by the child's performance in the probe tests you administered at the start of the **previous** session.

‐ If the child scores 4/4 on either of the explicit and/or implicit story sets which were taught previously, then they are ready to move on to a new story set (2a) **from the same therapy condition** in the next week's session.
‐ A child may be ready to move on from one story condition (implicit or explicit, signed vs. no sign) before the other, or at the same time.
‐ **After 2 sessions**, if a child still does not know 4/4 words from a story set, move onto Story b from the same condition (i.e., if you started with Story 1a in the Explicit Sign condition, then move to Explicit Sign Story 1b in the next session. If you were on Story 2a, then move to Story 2b, etc.). This will contain the same target words but within a different story to maintain the child's interest. Continue with this story for Sessions 3 and 4.
‐ **After 4 sessions,** if a child still does not know 4/4 words from a story set, then you should move onto the next story set from the same condition (i.e., if you started with Explicit Sign Story 1a, then moved to Explicit Sign Story 1b, introduce Explicit Sign Story 2a in the next session). This will contain different target words but will be presented the same way as for the first two stories.
‐ If the child has worked through all the story sets before the end of the therapy block (up to 16 sessions), you could go back and review some of the earlier story sets where the child had not learned all of the words.

Please ask if you are unsure which stories you should be working on. Remember, the decision re: which stories to target will be made *in between* sessions—you will already have planned which words you are working on for the current day at the end of the previous session.

## Appendix E

### Target Words and Age of Acquisition Ratings, Based on (Kuperman et al. 2012)

**Table:**

	Target word	Mean age of acquisition rating	Standard deviation
			
Verbs	Accomplish	7.84	2.75
	Agree	7.68	2.36
	Attach	6.79	1.75
	Attend	7.72	2.49
	Borrow	6.95	3.12
	Chuck	6.72	2.42
	Cling	7.74	3.38
	Dazzle	7.79	2.42
	Deny	7.8	2.04
	Explore	7.06	1.26
	Fail	6	1.61
	Fetch	6.61	2.55
	Manage	7.67	1.94
	Organise	6.33	2.09
	Participate	7.05	2.56
	Produce	7.75	3.11
	Protect	6.79	1.75
	Provide	7.74	2.62
	React	6.67	3.8
	Skedaddle	7.84	3.34
	Solve	6.58	1.92
	Spend	6.44	2.5
	Stroll	7.83	1.47
	Weep	8.5	2.7
Group average		**7.25**	**2.41**
			
Adjectives	Chilly	6.39	2.57
	Cruddy	6.88	3.3
	Deadly	6.11	1.78
	Dreadful	6.78	2.56
	Enormous	6.53	2.87
	Equal	6.47	1.58
	Floppy	6.68	5.09
	Fussy	6.67	2.2
	Greasy	6.39	1.85
	Heavenly	6.22	2.1
	Hilly	6	2.68
	Homemade	6.59	2.58
	Immediate	8.76	2.61
	Lazy	6.39	2.23
	Main	6.55	1.64
	Respectful	6.26	2.62
	Single	6.68	2.19
	Spend	6.44	2.5
	Strange	6.42	1.77
	Toothless	6.47	1.68
	Triple	6.78	1.44
	Useful	6.39	1.88
	Wild	6.11	2.51
	Wonderful	6.47	2.91
			
Group average:		**6.56**	**2.38**
			

## Appendix F

### Example Stories and Accompanying Images for Each Condition

**1) Reference condition**

*Listen out for the new words in the story and see if you can work out what they mean*

**The magic washing machine**

There was once a boy named Ethan who had an enormous love of horses. When he was 9, he got his own horse called Shadow. Shadow would cling to Ethan's side wherever he went. The boy and the horse had an equal love of exploring outside together.

One day, when Ethan was playing in his garden, he noticed something unusual—an enormous washing machine sitting in the corner. It was much bigger than any washing machine he had ever seen… Almost equal in size to Ethan's house!

Ethan decided to explore. He climbed onto Shadow's back, and together they trotted over to the washing machine. With equal fear and excitement, Ethan reached out to cling onto the enormous buttons. To his surprise, the washing machine began to hum loudly, as if it had come to life.

Shadow nudged Ethan with his nose, pushing him to climb inside the washing machine to explore some more. Ethan wasn't sure, but he always gave Shadow an equal chance to decide what to do. So, he bravely stepped inside. Soon, Shadow followed, clinging onto Ethan's hand.

They were amazed that the inside of the washing machine was like a whole new world—Inside, there was an enormous, colourful swirl of bubbles. Ethan and Shadow continued to cling onto each other tightly as they were whisked away on a whirlwind adventure.

They explored forests of hanging clothes and mountains of fluffy towels. The wet clothes seemed to cling to the air. They looked like an enormous sock puppet! Ethan and Shadow were feeling equal parts excited and amazed.

Eventually, the enormous washing machine began to slow down, and Ethan and Shadow found themselves back in their garden. They were still clinging to the memories of their washing machine adventure. But they knew there would be many new worlds and cultures to explore together in the future. Then, they began to feel very hungry. Ethan broke his sandwich into equal pieces to share with Shadow. After lunch, Ethan was keen to explore outside the garden. But when he turned to Shadow, he found that the horse was fast asleep!

**2) + explicit condition**

Pre‐story introduction

Let's listen to the words in our story today.

1. *Borrow* is a blue ‘doing’ word. It means to use something and then give it back again.

I could say: ‘Please can I borrow your rubber?’ That would mean ‘Please can I use your rubber and then I will give it back to you’.

*What is the new word?*

**If the child cannot remember a word, we will use the following cueing hierarchy: offer the child a phonemic cue. If they still can't retrieve the target then we say the word and ask them to repeat it back**.

2. Our next word is: *Cruddy. This is a green ‘describing’ word*. It means ‘dirty’ or unpleasant.

As in, ‘John's bedroom was *cruddy* because he didn't clean it’. That means that John's bedroom was dirty and unpleasant because he didn't clean it.

*What is the new word?*

**Use cueing hierarchy, if needed**

3. *Produce* is another blue ‘doing’ word. It means ‘to make or create something’.

As in, ‘The factory will produce thousands of toys for Christmas’ That means: ‘the factory will make thousands of toys for Christmas’.

*Can you remember the new word?*

**Use cueing hierarchy, if needed**

4. Our last new word is *Lazy*. This is a green ‘describing word’. It means ‘not wanting to work’ or use energy. Like when you can't be bothered to do something.

As in ‘The cat was too lazy to chase the mouse’. That means ‘the cat couldn't be bothered to run after the mouse’.

*What was the new word?*

**Use cueing hierarchy, if needed**

*Now listen out for the new words in our story*:

**School sports day**

One sunny day, the children of Stony House school were buzzing with excitement for their sports day. All except for Timmy. He was lazy and hated sport. He also hated his cruddy old trainers. His Mum bought them for his Birthday last year, but they had been in the cupboard ever since, gathering dust and grime.

As the day began, the children came together on the field. They were ready to start the different games and races but there was one problem: the sports equipment looked a bit cruddy. The balls had no air and the skipping ropes were broken. The teachers said that they would need to borrow some things from another school. But the nearest Primary was in the next town – at least an hour away from Stony House.

The children got together and decided they would make the best of what they had. They set out to produce the most fun‐filled sports day ever. First up was the sack race. The children used leftover sacks from the school coffee shop. Timmy's friend Saira was usually a bit lazy when it came to exercise. But she surprised everyone with her energy as she hopped and skipped towards the finish line.

Next came the egg and spoon race. The children borrowed their spoons and eggs from the school kitchen. Timmy managed to balance his egg all the way to the end without dropping it. Everyone produced a big cheer. They agreed that it had been the best sports day ever. They celebrated together and shared high fives. Then Timmy went home to rest and play on his Playstation.

Post‐story recap:

Let's think about the words in our story.

1. *Borrow* means to use something, then give it back.

I could say: ‘Dad borrowed a car from the garage when his was broken’ That would mean ‘Dad used a car from the garage until his one was fixed’.

2. Our next word was: *Cruddy*. This means ‘dirty or unpleasant.

As in, ‘His clothes became cruddy after a month of wearing them’. That means ‘his clothes got dirty and unpleasant because he wore them for a month’.

3. *Produce* means to make or create.

As in, ‘The bakery will produce fresh bread every morning for its customers’. That means: ‘The bakery will make fresh bread every morning for people to buy’.

*Can you remember the new word?*

4. *Lazy* means ‘not wanting to work or use energy’.

As in ‘The boy was too lazy to complete his homework’. That means ‘The boy did not want to do his homework because he couldn't be bothered’.

Which of our words means: ‘to use something, then give it back’?

Which word means ‘dirty or unpleasant’?

Which word means ‘to make or create’

Which word means: ‘not wanting to work’?

***If the child cannot remember a word, we will use the following cueing hierarchy: First, offer a phonemic cue. If they still can't retrieve the target then we say the word and ask them to repeat it back***.

**3) + sign condition**

*Let's listen to our next story now. Remember to watch the signs to help you work out what the new words mean*.

**The School trip** *Remember to sign all target words in the story*

In the last week of term, the children from Key Stage 2 were going on a school trip.

‘We have lots of children with allergies,’ said Miss Miles, the teacher. ‘Solomon reacts badly to peanuts and Adrian can't eat cheese. So I don't want to see a single nut or cheese sandwich in your lunchboxes. And I expect respectful behaviour at all times.’

When the school bell rang, the children chucked their reading books in their desks and lined up to get on the coach. There was a respectful silence as the teacher, Miss Miles, counted everyone. She wanted to make sure she did not lose a single child. The headmistress would not react well if anyone went missing. Miss Miles would probably get chucked out of the school!

When everyone was on the coach, the teacher reminded them to be respectful to the driver. ‘Please do not chuck a single thing; no pens, no balls or paper aeroplanes.’ The children reacted by smiling and giggling. It was not very respectful. But at least they didn't break any rules and they soon arrived at the outdoor activity centre.

The activity leader was called Carl. ‘Right everyone. Chuck your bags over there and put on a life jacket. Let's get started.’ Carl did not react when the children started messing about and teasing each other. He carried on speaking in a respectful voice and soon, the class calmed down. Then, they each got into their own single canoe and tried paddling along the river.

A few moments later, there was a loud cry. ‘Man overboard!’ shouted Miss Miles. Solomon had fallen into the water. ‘Quick – chuck him a lifebelt!’ Carl reacted quickly. He jumped into the water to rescue Solomon. Everything was fine. The children gave Carl a respectful round of applause to say, ‘Well done’. If you had been a single minute later, who knows what would have happened,’ said Miss Miles. ‘I’ glad it was only a single child,’ said Carl. ‘I was just doing my job.’

Soon, it was time for lunch. So the children chucked their life jackets onto a pile and sat down to eat together. The rest of the day went well, apart from when Adrian got stung by a bee. His skin reacted by coming out in a rash. But luckily, Miss Miles had some special cream which cleared it up in no time. At the end of the day, the children were still full of energy. But Miss Miles was exhausted and fell asleep on the coach back home.

**4) + explicit & sign condition**

Pre‐story introduction

Let's listen to the words in our story today.

1. *Spend (+sign)* is a blue ‘doing’ word. It means to pay money to buy something.

*Can you show me the sign?*

**If necessary, show the child the sign again and ask them to copy you**.

I could say: ‘My Mum is going to *spend (+sign)* £50 on a new coat’ That would mean ‘Mum is going to pay £50 for a new coat’.

*Can you show me the sign again?*

**If necessary, show the child the sign again and ask them to copy you**.

*What is the new word?*

**If the child cannot remember a word, we will use the following cueing hierarchy: ask them if they can do the sign. If not, SLT gives them the sign. If they still can't remember the word, the next step will be offering a phonemic cue. If they still can't retrieve the target, then we say the word and ask them to repeat it back**.

2. Our next word is: *Deadly (+sign). This is a green ‘describing’ word*. It means ‘makes something die’.

*Can you show me the sign?*

**If necessary, show the child the sign again and ask them to copy you**.

I could say: ‘That plant is *deadly (+sign)*’. That means ‘if a person or animal ate the plant, they would die’.

*Can you show me the sign again?*

**If necessary, show the child the sign again and ask them to copy you**.

*What is the new word?*

**Use cueing hierarchy, as necessary**

3. *Weep (+sign)* is another blue ‘doing’ word. It means ‘to cry’.

*Can you show me the sign?*

**If necessary, show the child the sign again and ask them to copy you**.

I could say: ‘The child began to *weep (+sign)* when she dropped her ice cream on the floor’ That means: ‘The child cried when she dropped her ice cream’.

*Can you show me the sign again?*

**If necessary, show the child the sign again and ask them to copy you**.

*Can you remember the new word?*

**Use cueing hierarchy, as necessary**

4. Our last new word is *Fussy (+sign)*. This is a green ‘describing word’. It means ‘picky’ or only liking things to be a particular way.

*Can you show me the sign?*

**If necessary, show the child the sign again and ask them to copy you**.

I could say: ‘The little girl was so *fussy (+sign)* about her clothes that she would only wear dresses with stripes’. That means ‘the girl was so picky about her clothes she would only wear stripy dresses’.

*Can you show me the sign again?*

**If necessary, show the child the sign again and ask them to copy you**.

*What was the new word?*

**Use cueing hierarchy, as necessary**

*Now listen out for the new words in our story and watch the signs. See if the signs help you understand and remember the new words*:

**Lily's Birthday party** *Remember to sign all the target words in the story.*

One Saturday, Lily woke up bright and early. It was her birthday, and she was very excited to celebrate with her friends. She had spent weeks decorating the house and was very fussy about how everything looked. She wanted everything to be just perfect for her big day. She had even persuaded her Mum to spend £10 on special balloons and party bags for all her guests.

Across the town, her best friend Jaden was getting ready for the party. He was trying to wrap Lily's present. But he kept getting the Sellotape stuck and ripping the paper. ‘Be careful with sharp scissors. They can be deadly if you're not careful’, said Mum. She always got so stressed about everything. It made Jaden want to weep!

Finally, it was time to go to the party.

On the way, Jaden and his Mum met their neighbour, Mrs. Oakley. She was weeping because she had lost her cat. Jaden wanted to help, but Mum said: ‘Be careful. There are deadly mushrooms in this garden. Let's hope the cat doesn't eat them’.

After a while, they found the cat hiding under Mrs. Oakley's apple tree. Luckily, he was a fussy eater and hadn't touched the poisonous mushrooms.

Finally, Jaden arrived at the party. He gave Lily her present and she opened it straightaway. It was a beautiful picture. ‘How much did you spend on this?’ asked Lily. Jaden didn't tell her that he had painted the picture himself. At that moment, Lily's Mum arrived with her Birthday cake. The friends sang ‘Happy Birthday’, ate cake and had lots of fun together.

Post‐story recap:

Let's think about the words in our story.

1. *Spend (+sign)* means to pay money to buy something.

*Can you show me the sign?*

**If necessary, show the child the sign again and ask them to copy you**.

I could say: ‘How much money did you *spend (+sign)* on my Birthday present?’ That would mean ‘How much did you pay for my Birthday present?’.

2. Our next word was: *Deadly (+sign)*. This means ‘makes something die’.

*Can you show me the sign?*

**If necessary, show the child the sign again and ask them to copy you**.

As in, ‘The poison was *deadly (+sign)*’. That ‘means if a person or animal drank the poison, they would die’.

3. *Weep (+sign)* means to cry.

*Can you show me the sign?*

**If necessary, show the child the sign again and ask them to copy you**.

As in, ‘The film was so sad it made me *weep (+sign)*’. That means: ‘The film was very sad so it made me cry’.

4. *Fussy (+sign)* means ‘picky’ or only liking things to be a particular way.

*Can you show me the sign?*

**If necessary, show the child the sign again and ask them to copy you**.

As in ‘The cat was very *fussy (+sign)* about its food so he would only eat fresh fish.’ That means ‘the cat was so picky that he would only eat fish’.

Which of our words means: ‘to pay’?

Which word means ‘makes something die’?

Which word means ‘cry’

Which word means: ‘picky’?

**If the child cannot remember a word, we will use the following cueing hierarchy: ask them if they can do the sign. If not, SLT gives them the sign. If they still can't remember the word, the next step will be offering a phonemic cue. If they still can't retrieve the target then we say the word and ask them to repeat it back**.

## Appendix G

### Target Definitions and Acceptable Alternatives **(based on results from Pilot project)**

**Table:**

Target word	Taught definition	Acceptable alternatives	Not accepted
Accomplish	Finish a task	To finish something, you did it	You've won something
Agree	Think the same	You accept it, they both say ‘yes’, when you both have the same answer	
Attach	Join together	Something goes together, connect	When something is on something
Attend	Be at something	Go to something	Get something, when I'm going to do something right now
Borrow	Use and give back	You ask to use it, get something then giving it back	Look after
Chilly	Cold		Something hot (if student gave this definition, they were prompted to say if they knew another meaning for chilly)
Chuck	Throw	Throw it away	
Cling	Hold onto	Another person hangs onto you	
Cruddy	Dirty		Very hard
Dazzle	To make blind for a short time	Sparkle, it shines in your eye	Shiny, when you wriggle something, very happy and jolly, being a bit dramatic, something beautiful
Deadly	Makes something die		It means it died and it looks very old.
Deny	Say no to something	Say that you didn't do anything, no,	Something you don't want to have
Dreadful	Very bad	Terrible, something bad is going on	You don't want to do it, when you're bossy
Enormous	Very very big	Really big, big, massive, giant, it's like this big (holds arms out wide)	
Equal	Same	Exactly the same, when the thing is the same thing, the same amount	Sharing out
Explore	Find out about	Search something, go to the places that are different or visit, you look all over, going on a little adventure	See something
Fail	Not pass	You haven't done something right, you did it wrong	Lose
Fetch	Go and get	When you get the thing who throwed it	Catch something, throwing a ball for a dog
Floppy	Soft and saggy	Like bendy, goes down (+ floppy gesture)	Moves around a lot
Fussy	Picky		Silly, you get mad, greedy
Greasy	Oily	It's like wet because you haven't washed it, something slimy	Slippery
Heavenly	Lovely		Something heavy
Hilly	With lots of hills	Very steep and bumpy	
Homemade	Made at home		
Immediate	Right now	Do it straight away, quick, as fast as you can	
Lazy	Not wanting to work	Sit down a lot; ask someone to get something for you when you can, you don't want to do anything (closes eyes), you're not bothered to do it	You're just bored, silly
Main	Most important	Important	Like a part of something
Manage	Be in charge of	You can do it on your own	Try to do it, you have to do it, in something, I'm trusted with that
Organise	Put in order	To set everything on your table tidy, put away, make it neatly, you've sorted in different places	
Participate	Take part in	To be in something, you're doing something with other people, joining something	When you want to do it, like in football
Produce	Make something		Provide something
Protect	Look after	Not let anyone get hurt, cover your clothes from getting dirty, standing up for your friend [if they are being bullied]	Help someone
Provide	Give		Share out, you find something then you give it to someone
React	Change after something	You move quickly, like if someone was going to attack you. You eat something and straight away start coughing	You do something quickly, when you haven't done something before
Respectful	Polite		I'm angry, I'm sad, when you're something kind, you believe in someone
Serious	Important		Grumpy all the time, you have done it but the other person doesn't believe you, being very dramatic, you're angry
Single	Only one	That fruit is by itself, it is one, all alone	The same as something
Skedaddle	Run away	Go on, just go out the door	
Solve	Work something out	You get the right pieces of the puzzle, figuring out something, you did the question correct	You found something that you lost
Spend	Pay money	Give some money, you're buying something, when you use your money to buy something	
Strange	Different	Something is weird, odd, something peculiar	
Stroll	Walk slowly	Walk, a little walk	
Toothless	With no teeth	Teeth out	You only have one tooth
Triple	Three		Double of something
Useful	Helpful	You can use it, something that helps you out	Kind, very good
Weep	Cry		
Wild	Lives outside the house	Some animals live in the forest	You're crazy (if student gave this definition, they were prompted to say if they knew another meaning for wild). You saw a tiger, I wouldn't touch it if I were you because it might poison you
Wonderful	Very good	Great, good, amazing	

## Appendix H

### Model Estimates

**Question 1**

**TABLE H1:** Estimates and odds ratios from the model fitted to the definition task data. The two probability columns (P) show the probability that a given parameter is linked with an *increase* in the outcome variable.

Fixed effect (baseline level)	Estimate [95% CI]	Est. error	*P*(*β*) > 0	Odds ratio [95% CI]	*p*(OR) > 1
+explicit (pre‐intervention)	−0.70 [−1.40;0.01]	0.35	0.03	0.77 [0.57;1.00]	0.03
+sign (pre‐intervention)	−0.54 [−0.93; −0.14]	0.20	0	0.81 [0.70;0.94]	<0.01
+explicit & sign (pre‐intervention)	−0.40 [–1.10;0.30]	0.35	0.12	0.87 [0.67;1.11]	0.11
post‐int. (reference)	0.67 [0.13;1.24]	0.28	0.99	1.18 [1.06;1.33]	0.99
post‐int. (+explicit)	NA	NA	NA	1.71 [1.48;1.99]	1
post‐int. (+sign)	NA	NA	NA	1.69 [1.47;1.95]	1
post‐int. (+explicit & sign)	NA	NA	NA	1.53 [1.35;1.76]	1
+explicit X post‐int.	1.12 [0.57;1.67]	0.28	1	1.11 [0.95;1.29]	0.91
+sign X post‐int.	1.23 [0.64;1.80]	0.29	1	1.17 [1.07;1.27]	1
+explicit & sign X post int.	0.98 [0.42;1.54]	0.29	1	1.12 [0.96;1.30]	0.93

**TABLE H2:** Estimates and odds ratios from the model fitted to the sentence generation task data. The two probability columns (*P*) show the probability that a given parameter is linked with an increase in the outcome variable.

Fixed effect (baseline level)	Estimate [95% CI]	Est. error	*P*(*β*) > 0	Odds ratio [95% CI]	*p*(OR) > 1
+explicit (pre‐intervention)	0.08 [−0.72;0.90]	0.41	0.48	1.06 [0.73;1.49]	0.58
+sign (pre‐intervention)	−0.20 [−0.64;0.22]	0.22	0.58	0.91 [0.74;1.10]	0.17
+explicit & sign (pre‐intervention)	−0.02 [−0.81;0.79]	0.41	0.18	1.01 [0.69;1.43]	0.48
post‐int. (reference)	0.68 [0.04;1.33]	0.33	0.98	1.30 [1.10;1.54]	0.99
post‐int. (+explicit)	NA	NA	NA	1.51 [1.29;1.78]	1
post‐int. (+sign)	NA	NA	NA	1.57 [1.31;1.87]	1
post‐int. (+explicit & sign)	NA	NA	NA	1.49 [1.26;1.76]	1
+explicit X post‐int.	0.49 [−0.13;1.12]	0.32	0.94	1.22 [0.92;1.58]	0.91
+sign X post‐int.	0.46 [−0.12;1.05]	0.30	0.94	1.09 [0.95;1.26]	0.89
+explicit & sign X post int.	0.39 [−0.22;1.00]	0.31	0.90	1.14 [0.86;1.49]	0.81

**Question 2**

**TABLE H3:** Estimates from the model fitted to the definition task data. Last column is the probability that a given parameter is linked with a *decrease* in the outcome variable.

Fixed effect (baseline level)	Estimate [95% CI]	Est. error	*P*(*β*) < 0
+explicit (pre‐intervention)	−0.29 [−0.74;0.17]	0.23	0.90
+sign (pre‐intervention)	−0.16 [−0.85;0.54]	0.35	0.67
post‐int. (+explicit & sign)	1.63 [1.07;2.17]	0.28	0
+explicit X post‐int.	0.16 [−0.40;0.72]	0.29	0.28
+sign X post‐int.	0.27 [−0.31;0.86]	0.30	0.19

**TABLE H4:** Estimates from the model fitted to the sentence generation task data. Last column is the probability that a given parameter is linked with a decrease in the outcome variable.

Fixed effect (baseline level)	Estimate [95% CI]	Est. error	*P*(*β*) < 0
+explicit (pre‐intervention)	0.10 [−0.32;0.50]	0.21	0.32
+sign (pre‐intervention)	−0.19 [−1.01;0.63]	0.42	0.67
post‐int. (+explicit & sign)	1.03 [0.43;1.64]	0.31	0
+explicit X post‐int.	0.11 [−0.52;0.74]	0.32	0.36
+sign X post‐int.	0.10 [−0.50;0.70]	0.31	0.38
