# Supplementary material for: Investigating the Effects of Adding Explicit Teaching and/or Signing to Story‐Based Vocabulary Intervention for School‐Aged Children With (Developmental) Language Disorder
Source: Int J Lang Commun Disord. 2026 Aug 31;61(5):e70317. doi: 10.1111/1460-6984.70317 (PMC13528867; doi:10.1111/1460-6984.70317)
Supplement: Supplementary file 1 [file JLCD-61-0-s002.docx]

**TIDieR Checklist: Story-based Vocabulary Intervention Programme (Story-VIP+), with or without explicit instruction and/or iconic sign**

| **TIDieR Item** | **Description** |
| --- | --- |
| 1. Brief Name | **STORY-VIP+**  **Full name:** *Story-based Vocabulary Intervention Programme, with or without explicit instruction and/or iconic signing.* |
| 2. Why (Rationale, Theory, Goal) | Improve understanding and retention of new words, listening comprehension and vocabulary use, supported by story context, explicit teaching and/or iconic signing. Theory: Multiple exposure in different contexts, explicit instruction and iconic signing can enhance word learning through different mechanisms, e.g. cross-situational generalization; compensating for difficulties with implicit learning and dual coding hypothesis. |
| 3. What (Materials) | Intervention session plans and record sheets; Story videos (tailored to each teaching condition); Written script for introduction and recap (*+ explicit* and *+ explicit & sign* conditions); Sign videos (*+ sign* and *+ explicit & sign* conditions); Child rating scale |
| 4. Procedures | **Condition assignment:** This is randomised and alternates between sessions (either all words are signed within a session, or none). Two stories are presented per session: (one with and one without explicit teaching, either signed or not signed).  **Initial session**: Explain rationale; ask child to complete self-rating scale; watch story videos; for *+ explicit* and *+ explicit & sign* conditions, introduce target words and recap after story presentation; apply feedback hierarchy as directed in the session plan for each condition.  **Subsequent sessions:** Quickfire recap; decide progression based on probe scores from the previous week; alternate conditions and follow the procedures for story presentation and feedback from above.  **Final session:** As above. At the end of the session, ask child to repeat self-rating scale |
| 5. Who Provided | The child’s usual speech and language therapist (SLT). |
| 6. How | Face-to-face, individual sessions using structured story-based activities. |
| 7. Where | Quiet room within the child’s school. |
| 8. When and How Much | Up to 16 sessions; twice a week for half an hour. Each session includes two stories; each target word is repeated 6 times per session. |
| 9. Tailoring | Progression based on child’s performance on probe tests at the start of each session. |
| 10. Modifications | Change from story a) to story b), containing the same target words, if child does not achieve mastery after 2 sessions. After 4 sessions, move onto the next target word set within the same condition, irrespective of whether the child has achieved mastery or not. |
| 11. How Well (Fidelity) | SLT monitors exposure count (6 per word). Session notes used for attendance records and fidelity checks. |
| 12. Planned/Actual | Document planned vs actual delivery in session record sheets. |
